# Primary eye health services for older adults as a component of universal health coverage: a scoping review of evidence from high income countries

**DOI:** 10.1016/j.lanwpc.2022.100560

**Published:** 2022-08-12

**Authors:** Lucy Goodman, Lisa Hamm, Benoit Tousignant, Joanna Black, Stuti Misra, Sophie Woodburn, Lisa Keay, Matire Harwood, Iris Gordon, Jennifer R. Evans, Jacqueline Ramke

**Affiliations:** a School of Optometry & Vision Science, Faculty of Medicine and Health Sciences, The University of Auckland, 85 Park Road, Grafton, Auckland 1023, New Zealand; b School of Optometry, Université de Montréal, 3774 Jean-Brillant, Montreal, Quebec H3T1P1, Canada; c School of Public Health, Université de Montréal, 3774 Jean-Brillant, Montreal, Quebec H3T1P1, Canada; d Department of Ophthalmology, New Zealand National Eye Centre, Faculty of Medical and Health Sciences, The University of Auckland, 85 Park Road, Grafton 1023, Auckland, New Zealand; e Specsavers Queenstown, 12 Hawthorne Drive, Frankton, Queenstown 9300, New Zealand; f School of Optometry and Vision Science, UNSW Sydney, Rupert Myers Building (M15), Southern Dr, Kensington, NSW 2052, Australia; g The George Institute for Global Health, UNSW Sydney, Level 5/1 King St, Newtown NSW 2042, Australia; h Department of General Practice & Primary Health Care, School of Population Health, University of Auckland, 28 Park Ave, Grafton Auckland 1023, New Zealand; i International Centre for Eye Health, London School of Hygiene & Tropical Medicine, Keppel St, London WC1E 7HT, United Kingdom; j Centre for Public Health, Institute of Clinical Science, Block A, Royal Victoria Hospital, Queen's University Belfast BT12 6BA, United Kingdom

**Keywords:** Universal health coverage, Healthy ageing, Older people, Eye health service

## Abstract

In pursuit of Universal Health Coverage (UHC) for eye health, countries must strengthen services for older adults, who experience the highest prevalence of eye conditions. This scoping review narratively summarised (i) primary eye health services for older adults in eleven high-income countries/territories (from government websites), and (ii) the evidence that eye health services reduced vision impairment and/or provided UHC (access, quality, equity, or financial protection) (from a systematic literature search). We identified 76 services, commonly comprehensive eye examinations ± refractive error correction. Of 102 included publications reporting UHC outcomes, there was no evidence to support vision screening in the absence of follow-up care. Included studies tended to report the UHC dimensions of access (*n=*70), equity (*n=*47), and/or quality (*n=*39), and rarely reported financial protection (*n=*5). Insufficient access for population subgroups was common; several examples of horizontal and vertical integration of eye health services within the health system were described.

**Funding:**

This work was funded by Blind Low Vision New Zealand for Eye Health Aotearoa.

**Research in context**

**Evidence before this study**

In its inaugural *World Report on Vision* in 2019, the World Health Organization called for eye care to be included in efforts to achieve Universal Health Coverage (UHC). In 2021, the *Lancet Global Health* Commission on Global Eye Health supported this call, stating that UHC should not be considered “universal” without including eye health. As the prevalence of eye conditions and vision impairment increases with age, older adults are an important group to consider when strengthening eye health services.

In 2020, the government of Aotearoa New Zealand (hereafter ‘New Zealand’) proposed funding eye health checks for the population ≥65 years. This proposal aligned with New Zealand's *Healthy Ageing* strategy, but how this eye health check should be implemented was unclear. In July 2020, we conducted a preliminary search of the published literature within the PubMED database (using “vision screening” and “New Zealand” MeSH search tags), where we observed a paucity of evidence describing adult eye screening services in New Zealand. Widening our search internationally, we identified three systematic reviews (from Cochrane, and both the Canadian and US Preventative Task Force), each reporting insufficient evidence that community vision screening services for older people (such as the proposed “eye health check”) can reduce vision impairment in this population.

Countries such as New Zealand that are attempting to improve eye health among older adults are challenged by the lack of evidence supporting the effectiveness of primary eye health services, and the best way to structure these services within the health system to achieve UHC. We designed this review to fill this evidence gap, and therefore to better inform future eye services in New Zealand and elsewhere.

**Added value of this study**

This review provides the practical and theoretical evidence that can direct policy makers in high-income countries towards achieving UHC when designing new eye health services for older people. Firstly, we collated the types of primary eye health services available in New Zealand and comparable high-income countries (Australia, the devolved countries of the United Kingdom, Ireland, Singapore, Hong Kong, Canada, and the United State of America). A key finding was that community vision screening services for older adults were uncommon (we identified only one visual acuity screening service, in Singapore). All other included countries, except New Zealand, funded eye examinations (often with refractive error correction) for all older adults, or a subgroup considered most at risk of financial barriers. Secondly, we systematically scoped the literature for evidence of services that reduced population vision impairment or incorporated UHC in its design and implementation (access/coverage, equity, quality [using the Institute of Medicine (IOM) dimensions: integration, efficiency, timeliness, safety, and people-centredness], or financial protection). The identified evidence describes a need for services that prioritise underserved population groups, to avoid further widening existing inequalities (e.g. as observed in Scotland's universal eye care policy); a lack of evidence reporting financial protection for health system users; and several examples of service integration (e.g. shared care schemes) that may improve access to services via horizontal and vertical integration.

**Implications of all the available evidence**

Vision screening for older adults (in isolation from broader eye health services) was used in only one of the countries we assessed, and there is no evidence to suggest this strategy reduces vision impairment. Financial protection is a cornerstone of UHC and all countries except New Zealand provided this protection for at least some older adults. We identified a range of strategies that countries can consider when wishing to improve access, quality, or financial protection of primary eye health services for older adults as part of UHC initiatives. Embedding equity into new strategies by prioritising the needs of the historically underserved groups will help avoid intervention-generated inequities.

Alt-text: Unlabelled box

## Introduction

In its inaugural *World report on vision*, the World Health Organization called for eye health to be part of efforts to achieve universal health coverage (UHC) through implementing Integrated People-centred Eye Care (IPEC) across the spectrum of promotive, preventative, curative and rehabilitative services.^1^ In 2020 this call was adopted by 194 countries at the 73rd World Health Assembly.^2^ UHC is defined as *people being able to access the care they need, of sufficient quality to be effective, without suffering financial hardship*.^3^ UHC has received increasing attention in eye health in recent years, including as a central theme of the *Lancet Global Health* Commission on Global Eye Health.^4^

Eye conditions are strongly associated with increasing age—more than three-quarters of the estimated 43 million people living with blindness and almost two-thirds of the 553 million with distance vision impairment in 2020 were aged ≥50 years.^4^ Older adults may face a range of barriers to accessing health services that prohibit healthy ageing.^5^ Therefore, when countries are planning to improve access to eye health for their population, older adults are a group in need of particular attention. In 2020, the government of Aotearoa New Zealand (hereafter called New Zealand) announced a strategy to provide eye health checks to the population ≥65 years.^6^ However, New Zealand had minimal research on eye health services or monitoring data to inform the government on which strategy would be most appropriate and effective to improve access to eye health services for older New Zealanders.^7^

The aims of this review were to summarise (i) the nature and extent of community or primary eye health services for older adults in eleven high-income countries and territories, and (ii) the extent to which eye health services in these settings reduce vision impairment and/or provide UHC of eye health services for older adults. While the lack of synthesized evidence in New Zealand provided the impetus for this review, we believe the findings are relevant for many countries wishing to improve access to eye health services in pursuit of UHC. We have synthesised our findings as a scoping review due to the broad nature of the research questions and the diversity of the included evidence.

## Methods

### Overview

The protocol^8^ was registered on the Open Science Framework on 31^st^ October 2020, following input from a stakeholder group.

We looked for evidence in the grey and published literature by asking two complementary questions.

**Question 1: Existing eye health services**

What government-led community or primary eye health services for older adults are offered in New Zealand and similar countries, and how are they structured within the health system?

**Question 2: Availability of evidence**

What is the evidence that eye health services within the selected countries:

i) reduce vision impairment? and/or
ii) provide UHC of eye health for older adults?

We envisaged limited evidence reporting outcomes on vision impairment from the specific services identified in the grey literature. Therefore, we sought evidence that described services in the included countries across any of the UHC dimensions of access, quality, equity, or financial protection, with the assumption that services addressing these dimensions are contributing to strengthening eye health services.

### Search strategy & selection criteria

For both Questions 1 and 2, the included evidence reported eye care services within eleven countries: New Zealand, Australia, Canada, Ireland, United Kingdom (England, Scotland, Wales and Northern Ireland), Singapore, Hong Kong, and the United States of America. The included countries were predominantly English-speaking high-income countries or territories^9^ with a population of at least 2 million^10^ in 2019 considered to have health care systems with similarities to that in New Zealand. The included countries also ranked relatively highly in their health expenditure per capita,^11^ and the WHO's UHC service coverage index^12^ (Supplementary Table 1).

We used separate but complementary inclusion criteria to address the two questions (Table 1).

**Table 1:** Inclusion or exclusion criteria for evidence addressing Question 1 and 2.

Characteristics of service/evidence	Question 1: Existing eye care services	Question 2: Evidence for the effectiveness of eye care services
**Population**	• Older adults (∼65 years and above), or adult populations where outcomes for older adults are described separately.	
**Setting**	• Services or evidence from ≥1countries most relevant to the New Zealand health system (defined as high-income countries^9^ with ≥2 million^10^ population in 2019, where English is an official language): New Zealand, Australia, Canada, Ireland, United Kingdom (England, Scotland, Wales, Northern Ireland), Singapore, Hong Kong, and the United States of America.	
**Time period**	• Available/advertised services in mid-2020	• Evidence published between1 January 2010 and October 2020
**Interventions**	• Community or primary eye care services that offer screening, general eye care, treatment, referral, or rehabilitation, and report on at least one of the WHO building blocks (Question 1) or UHC dimensions (Question 2) described below.	
	• Administered at a state/provincial or national level • Wholly or partially funded by the government or other public funds *Exclusion criteria:* • Non patient-facing services, e.g. funding for research or equipment. • Research, pilot, time-limited, and/or non-governmental projects • Secondary or tertiary eye care services offered exclusively outside the primary care setting (e.g. hospital eye care services)	• Evidence relating to service delivery (specific or general eye care service) and/or: • Interventions that treat any of the eye conditions commonly causing vision impairment in older adults: cataract, uncorrected refractive error, age-related macular degeneration, glaucoma, or diabetic retinopathy *Exclusion criteria:* • Methodological evidence (e.g. technical comparisons, diagnostic accuracy of screening equipment).
**Types of evidence**	• Evidence written in English with full-text available.	
	• Information describing service structure retrieved from government web pages and relevant policy documents within, including reports, guidelines, audits, or government legislation. *Exclusion criteria:* • Outdated documents replaced by more recent information	• Experimental, quasi-experimental and observational studies, systematic and scoping reviews, overviews of systematic reviews, research letters • Grey literature describing service performance identified within Question 1 *Exclusion criteria:* • Qualitative research including case studies, opinions, editorials • Data derived solely from computer modelling (e.g. cost-effectiveness)
**Outcomes**	1. Service structure described by at least one of WHO's health system building blocks^63^: I. **Service delivery**: populations eligible for the service. II. **Medical technologies**: products and services offered: *Assessments*: eye examinations (standard/comprehensive or partial/follow-up examinations, urgent eye care, or diabetic eye examinations), screening (for general eye health, diabetic retinopathy, or glaucoma), and needs assessments (for rehabilitation). *Treatments*: refractive error correction, surgery or other ophthalmic services, low vision services, and prosthetic eye services. III. **Health workforce**: health practitioners who provide the service: Optometry clinician (optometrist/optician, dispensing optician), ophthalmology clinician (ophthalmologist, ophthalmic medical practitioner), technician/screener/nurse, specialty worker (e.g. rehabilitation worker, orthoptist, ocularist), general practitioner/physician. IV. **Health financing**: financial protection for service users, funding structure, management of finances between funder and provider, and. V. **Leadership and governance**: are those accountable for the service reported? VI. **Health information**: are standards for measuring service performance published, and is the service audited?	1. Change in population vision impairment 2. Eye care service described by at least one of the following UHC domains^64^: I. **Access**: service coverage or attendance II. **Equity**: differences in service provision between populations, or underserved populations. III. **Financial protection**: protection from, or burden of, service costs IV. Quality* (using the Institute of Medicine [IOM] domains^65^): ***Integration***: attempts to coordinate care between providers ***Efficiency***: processes to maximise available resources ***Timeliness***: measures of timely service delivery ***Safety***: personal safety for the service user ***People centredness***: prioritises service users’ needs and preferences *the remaining two IOM domains included above: *equity* as its own UHC domain and *effectiveness* as change in vision impairment) *Exclusion criteria:* • Self-reported vision impairment

For Question 1, one reviewer (LG) searched grey literature (including policy documents, reports, guidelines, audits, and general information described within government web pages) during August-October 2020, with verification by a second reviewer (SM, LK, BT). Eye care programmes were identified from the national government's website of each included country. Additional searches of state/provincial government websites were also completed, except for the USA, where the large number of states and the complexity of the health system meant only national-level services were included. Parallel programmes available within the devolved countries of the United Kingdom were included separately. General search terms, including “eye”, “vision”, “optical”, “optometry” and “ophthalmology” were used to identify eligible information within each website, and we repeated the search using the Google search engine. Relevant links within documents to other sources of information were pursued. Searching continued until retrieved results were unambiguously irrelevant.

For Question 2, an Information Specialist from Cochrane Eyes and Vision (IG) conducted a search in MEDLINE, Embase, Cochrane Library and the CRD Database (DARE, NHS EED and HTA) on 19^th^ October 2020 (Supplementary Table 2). Grey literature from Question 1 that described programme performance were also included. Screening of retrieved publications was conducted in Covidence (Veritas Health Innovation, Melbourne, Australia. Available at www.covidence.org). During each round of screening, two reviewers (from LG, JB, SM, BT, SW and JR) independently screened i) each title and abstract, then ii) the full-text manuscript of each potentially relevant publication, against the eligibility criteria (Table 1). Data extraction was performed in duplicate for ∼20% of included full text publications (*n=*20 publications). At each stage of screening, differences of opinion were resolved by discussion. The remaining data extraction was performed by a single reviewer (LG).

### Data synthesis

Primary eye care programmes included in Question 1 were described using the six WHO health system building blocks outlined in our study protocol^8^ and Table 1. Where relevant, programmes were assigned to multiple sub-categories within a building block. The number of programmes fulfilling each building block was calculated, and relevant examples described narratively.

For the evidence describing effectiveness of eye care programmes (Question 2), general information was extracted from each publication and included the country in which the data were collected, the study design, the year of publication, and the eye condition assessed or treated by the service. For the latter category, publications describing vision impairment in general (i.e. not a specific condition) were classified under ‘general’ eye conditions. For each publication, we summarised outcomes that described (i) change in vision impairment, and/or (ii) any of the UHC dimensions (access, equity, financial protection, or quality (integration, efficiency, timeliness, safety, and people-centredness)^8^ (Table 1). The number of included publications that reported each outcome was calculated, and the overall findings described narratively. All study types (i.e. observational and interventional studies) were analysed together, and evidence reporting specifically on eye care programmes identified in Question 1 was highlighted.

### Changes to the protocol

Our original intention was to complete a rapid systematic review that could inform New Zealand policy.^8^ However, due to the heterogeneity of the included evidence, we synthesised our findings as a scoping review and omitted the quality appraisal so that we could draw conclusions from a wider range of sources.

### Role of the funding source

The funders had no role in the study design, collection, analysis, or interpretation of data for this review.

## Results

### Question 1: Existing services for older adults

From our website search, we identified 76 eye care programmes relevant to older adults across the eleven included countries/territories. The service structure of the identified programmes across WHO's building blocks are summarised below (and in Table 2), and key examples of individual programmes are highlighted in Table 3. Most programmes from Australia and Canada were operated at a state or provincial level (12/15 and 29/31 programmes respectively), whereas included programmes from the remaining countries were nationally operated (Table 2).

**Table 2:** Characteristics of eye care programmes relevant to older adults in New Zealand and similar countries described within WHO building blocks.

SERVICE STRUCTURE			n (*N=*76)	%
**Country**	Canada	*national*	*2*	*2.6*
		*provincial*	*29*	*38.2*
	Australia	*national*	*3*	*3.9*
		*state*	*12*	*15.8*
	UK	*England*	*3*	*3.9*
		*Northern Ireland*	*3*	*3.9*
		*Scotland*	*2*	*2.6*
		*Wales*	*4*	*5.3*
		*Great Britain*	*1*	*1.3*
	USA		5	6.6
	New Zealand		4	5.3
	Ireland		3	3.9
	Singapore		3	3.9
	Hong Kong		2	2.6
**Service delivery**				
**Eligible population**^a^	Socially disadvantaged		33	43.4
	Condition-specific		26	34.2
	Older people		18	23.7
	General population		10	13.2
**Medical technologies**				
**Assessments**^a^	Eye examinations		42	55.3
	Diabetic retinal screening		9	11.8
	Glaucoma screening		3	3.9
	Visual acuity screening		1	1.3
	Other screening		1	1.3
**Treatments**^a^	Refractive error correction		44	57.9
	Low vision rehabilitation		13	17.1
	Prosthetic eye		8	10.5
	Surgery		8	10.5
**Health workforce**				
**Provider**^a^	Optometry clinician		55	72.4
	Ophthalmology clinician		37	48.7
	Technician / screener / nurse		9	11.8
	Specialty worker		6	7.9
	General practitioner		4	5.3
	Not identified		10	13.2
**Location**^a^	Healthcare locations		35	46.1
	Community locations		10	13.2
	Not identified		37	48.7
**Health financing**				
**Financial protection**^a^	Subsidised		55	72.4
	No out-of-pocket payment		20	26.3
	Loan		1	1.3
**Management of finances**^a^	Direct payment / reimbursement		71	93.4
	Voucher		8	10.5
	Not applicable		1	1.3
**Funding structure**^a^	Primarily government funded		74	97.4
	Public-private funding		2	2.6
**Leadership & governance**				
**Accountability**^a^	Governance identified		14	18.4
	Stakeholders identified		8	10.5
	Not identified		59	77.6
**Health information**				
**Reporting**^a^	Health monitoring identified		10	13.2
	Service performance identified		17	22.4
	Not identified		55	72.4

a As each programme can fulfil multiple categories within each building block, the total number of programmes may sum to >76.

**Table 3:** Examples of services relevant to older adults from countries of interest. Service structure is described within WHO Building Blocks and evidence from the literature across Universal Health Coverage dimensions.

Service	Service structure (WHO health system building blocks)	Universal Health Coverage dimensions reported in the published literature
**Victorian Aboriginal Spectacle Subsidy Scheme (Australia)**^66^	• *Service delivery*: Refractive error correction for Aboriginal and Torres Strait Islander people living in Victoria State. Available every two years. • *Medical technologies*: Spectacles with single vision, bifocal, or multifocal lenses. • *Health workforce*: Provided via a network of participating optometry practices and Aboriginal Health Service clinics. • *Health financing*: Government funded. Subsidised service (co-payment: AU$10/∼US$7). • *Leadership & governance*: Victorian State Government Department of Health and Human Services Aboriginal Health and Well-Being Branch, and the Australian College of Optometry. • *Health information*: Service audit 2016.^35^	• *Access:* Providing visual aids improves access to eye examinations (∼11,000 visual aids dispensed by 2016, and annual eye examinations increased from ∼300-350 during 2008-2010, to approximately 3,200 by 2016).^35^, ^36^, ^37^ • *Integration*: Providers face some challenges participating in the scheme (e.g. financial and time stressors), and service delivery (e.g. types of providers) varies between rural locations depending on the local opportunities and challenges.^35^ • *People-centredness*: Some practices are not yet culturally safe, and the service would benefit from greater stakeholder input (e.g. to improve the selection of available frames).^35^ • *Equity*: Overall, the scheme has a positive impact on the Aboriginal community that extends beyond eye health.^35^
**Equipment for people who are blind or have reduced vision (New Zealand)**^67^	• *Service structure:* Low vision aids for people with reduced vision. • *Medical technologies:* Mobility equipment: includes mobility canes, screen-reading software, or magnifiers. Glasses: low-vision eligibility criteria apply. • *Health workforce:* Low vision assessors (optometrist, eye specialist, or service coordinator for the Blind Foundation) recommend eligibility. • *Health financing:* Government funded. May be provided to user free of charge. • *Leadership & governance:* Coordinated by the Ministry of Health. • *Health information:* Stocktake and needs analysis 2015.^23^	• *Access:* Services are provided by six specialised low vision clinics offering free consultations, and a number of private optometrists offering fee paying services.^23^ • *Financial protection:* Most low vision aids are affordable, but some are expensive.^23^ • *Equity: T*here is a shortage of available services to meet the demand, particularly for Māori and Pacific people, and/or those living in provincial and rural areas.^23^
**National Health Service General Ophthalmic Services (UK)**^18^^,^^68^, ^69^, ^70^	• *Service delivery*: National service available every two years (England, Northern Ireland, Wales) or annually (Scotland) to those >60 years of age. • *Medical technologies:* Eyesight tests (England, Northern Ireland, Wales) or comprehensive ± supplementary eye examinations (Scotland), and optical vouchers for refractive error correction. • *Health workforce:* Provided via community eye care services (e.g. optometrists, ophthalmic practitioners, ophthalmologists, and dispensing opticians). • *Health financing:* Government funded. Free eye examinations with subsidised refractive error correction (optical voucher values: £39.10 to £215.50 / ∼US$53 to 292). • *Leadership & governance:* Co-ordinated by the National Health Service (England, Scotland, Wales) or the Health and Social Care Board (Northern Ireland). • *Health information:* Service statistics (England 2019/20,^71^ Northern Ireland 2019/20,^17^ Scotland 2018/19,^72^ Wales 2018/19^20^).	• *Access:* Across all countries, the number of people accessing vision tests and optical vouchers has increased over the last ∼decade (England: 38.2% between 2002/03 to 2019/20^71^; Northern Ireland: ∼13% over the decade to 2019/20^17^; Scotland: 47% between 2006/07 and 2018/19^72^; Wales: 10.6% between 2008/09 and 2018/19^20^), and domiciliary visits were approximately 3% of all sight tests.^17^^,^^20^^,^^71^^,^^72^ Most people accessing sight tests were aged 65 years and older (e.g. Northern Ireland: 67% aged <16 or >65 years in 2019/20^17^; Wales: 52.4% of tests in 2018/19^20^). More people accessed eye examinations when they were freely available to the general population (i.e. in Scotland, people accessing eye examinations increased from 32.1% in 2005 to 37.7% in 2006 when the free eye examination policy was introduced).^21^ • *Integration*: Most patients attending an eye appointment were managed within community optometry without referral to hospital eye services (e.g. Scotland: 95% managed in the community).^72^ • *Equity:* Access to vision tests was higher with lower levels of deprivation (e.g. Northern Ireland: for ages 60+: 71% in the most deprived vs 80% in the least deprived^17^) and varied between different regions (Northern Ireland,^17^ Wales^20^). People with lower education or income were less likely to take advantage of free eye examinations compared to more advantaged people (Scotland).^21^
**Eye Health Examination Wales (UK)**^73^	• *Service delivery:* National service available to people requiring urgent eye care. • *Medical technologies:* Comprehensive eye health examination. • *Health workforce:* Provided via community optometrists or ophthalmic practitioners registered with the scheme. Patients who require further treatment are referred to GP for general health conditions or to hospital eye services. • *Health financing:* Government funded. Provided to user free of charge. • *Leadership & governance:* Co-ordinated by the National Health Service. • *Health information:* Service statistics 2018/19.^20^	• *Access:* In 2018-19, over half (56%) of claims were for patients aged 60 years or over.^20^
**Northern Ireland Primary Eyecare Assessment and Treatment Service (NI-PEARS) (UK)**^74^	• *Service delivery*: National service available to people requiring urgent eye care. • *Medical technologies:* Eye examinations (and treatment where appropriate) for people requiring urgent or medically necessary eye care. • *Health workforce:* Provided via community optometrists. Patients who cannot be managed within the service are referred to GPs or hospital eye services. • *Health financing:* Government funded. Provided to user free of charge. • *Leadership & governance:* Coordinated by the Health and Social Care Board. • *Health information:* Service statistics 2019/20.^17^	• *Integration:* In 2019/20, 66% of new assessments were managed within the service or discharged with advice, and only 13% required urgent or routine referral to hospital eye services.^17^
**National Health Service Diabetic Eye Screening Programme (UK)**^75^, ^76^, ^77^, ^78^	• *Service delivery:* National retinal screening service for people with diabetes aged 12 years and older. Screening is offered annually and provided locally within national standards. • *Medical technologies*: Visual acuity test, digital photographs of both retinas (with dilation), slit-lamp examination if required. • *Health workforce:* Screening delivered locally by National Health Service and private providers: includes, screener, grader, screener grader, optometrist. Patients with detected eye disease are referred to digital surveillance clinic or hospital eye services for follow-up examinations and treatment. • *Health financing:* Government funded. Provided to user free of charge. • *Leadership & governance:* Co-ordinated by Public Health England, Northern Ireland, Scotland, and Wales. • *Health information:* Service statistics: England 2019/20,^79^ Northern Ireland 2016/17,^80^ Scotland 2018/19,^81^ and Wales 2018/19.^20^^,^^82^	• *Access:* Uptake of retinal screening: England: 82.3% in 2019-20^83^; Northern Ireland: 69.2% in 2016-17^80^; Scotland: 73.8% in 2018-19^81^ and 91.4% between 2005 and 2010^84^; Wales: 67.5% in 2018-19.^82^ • *Timeliness:* Screening offered within a timely manner (Northern Ireland: screening conducted every 13 months on average^80^; Scotland: 53.8% screened within one year of diagnosis^84^; Wales: 48.7% screened in 2018-19^82^). Results letter issued within three weeks (England: 97.1%^83^; Scotland: 92.2%^81^). Urgent follow-up with hospital eye services or digital surveillance within a timely manner (England: 74.2%^83^; Wales: 87.5%).^82^ • *Efficiency:* Screening efficiencies improved after the initial years of the programme (e.g. England: patients with diabetes diagnosis <1 year increased from 18.7% in 2008 to 48.6% in 2011^39^; Scotland: rates of referable eye disease were high during the initial years of the programme (7.0% and 6.0% in 2006 and 2007 respectively), and then stabilised (4.3% during 2008-2010).^41^ The median time to first retinal screening for those diagnosed in 2005 was 540 days (IQR 258-747), but this reduced to 83 days (IQR 51-135) by 2008).^84^ • *Safety*: Patient outcomes could be affected by poor image quality (Northern Ireland: 5.7% in 2017.^80^) • *Equity:* Variation in service coverage between population groups (Northern Ireland: variation between locations and across age groups (patients aged 61-70 most likely to attend).^80^ Wales: differences in coverage between most and least deprived areas was 9.8%.^82^; Scotland: some people experienced a longer delay between diagnosis and screening^84^).
**Welsh Low vision Service (UK)**^85^	• *Service delivery*: National low vision service for people with vision impairment or low vision, or who are registered as vision impaired. One needs assessment offered annually, and vision aids are provided for as long as required. • *Medical technologies:* Free low vision aids (including magnifiers, typoscopes, task lights, electronic magnifiers, shields, and/or reading stands). • *Health workforce:* Provided by low vision optometrists, ophthalmic medical practitioners, dispensing opticians with specialist training. Referrals to other support services, GPs, or ophthalmologists if required. • *Health financing*: Government funded. Provided to user free of charge. • *Leadership & governance:* Co-ordinated by the National Health Service Wales. • *Health information:* Service statistics 2018/19.^20^	• *Vision impairment:* The service improved visual acuity with low vision aids (pre: N12, inter-quartile range [IQR] N8-N24 vs 3-months post-service: N5, IQR N4-N6).^86^^,^^87^ • *Access*: The service increased number of low vision appointments (51.7% increase in one year prior vs one year post-intervention).^86^ The number of assessment performed increased between 2017-18 to 2018-19, and 65.9% were for patients aged 80 years or older.^20^ • *Integration*: No differences in patient centred outcomes (self-reported vision disability, patient satisfaction, or use of low vision aids) or clinical outcomes (change in visual acuity with low vision aids) between hospital or community based low vision services.^87^ Participants attending community low vision services were given more low vision aids than those attending hospital services (median, range: 3, 1-8 and 2, 1-6 respectively).^87^ • *Timeliness*: The service reduced waiting times (pre: 50% of people waited ≥6 months vs one year post-intervention: 60% <2 weeks).^86^ • *People-centredness*: The service reduced return journey travel time to the nearest service provider (by 16.50, IQR 1.66-39.32 minutes), and 98% of people found the service helpful.^86^ Patient satisfaction with the service was reduced at 18 months compared to 3 months after the service was initiated, although use of low vision aids (i.e. frequency) was no different between the two time points.^86^^,^^88^
**Diabetic Retina Screen (Ireland)**^89^	• *Service delivery*: National diabetic retinopathy screening service for people with diabetes aged 12 years and older. Patients are recalled annually from a national register. • *Medical technologies:* Screening: digital retinal photography (with dilation), slit lamp examination performed if necessary. Follow-up treatments: intravitreal injections, photocoagulation, vitrectomy. • *Health workforce:* Screening is provided at dedicated photography and grading centres, and is delivered by a team of trained screeners, clinicians, nurses, and administrative staff. Patients are referred to ophthalmologists within the service for treatment of retinopathy, maculopathy, or non-diabetic eye disease. • *Health financing:* Government funded. Screening and all necessary treatment and follow-up provided to user free of charge. • *Leadership & governance:* Co-ordinated by the National Screening Service. • *Health information*: Quality assurance standards in place for all aspects of screening. Service statistics published 2013-15,^89^ 2016-17,^43^ and 2018-19.^44^	• *Access*: Screening rates increased since the programme was initiated (from 46.8% in the first year to 69.7% in the fourth year).^42^, ^43^, ^44^ Between 2013-15, 63% of patients registered with the programme attended their screening appointment.^90^ • *Efficiency*: Referral rates have decreased since the programme was initiated (from 2.9% in the first year to 0.9% in the fourth year), illustrating the larger impact of detected retinopathy in the initial stages of the programme.^42^, ^43^, ^44^
**Primary Care Networks (Singapore)**^91^	• *Service delivery:* Retinopathy screening for diabetic patients through the Primary Care Network (PCN). Patients are enrolled in chronic disease registry and screened periodically. • *Medical technologies:* Retinal photography. • *Health workforce*: PCNs consist of private general practitioners (GPs) supported by nurse counsellors and care coordinators. GPs refer patients to ancillary screening services (e.g. at Community Health Centres), facilitated by the PCN coordinator, and results are sent back to GP for review and follow-up. • *Health financing***:** Government funded. Service users are charged a fee for screening, but a subsidy can be applied with eligible seniors’ health cards. • *Leadership & governance:* Ministry of Health Singapore. • *Health information:* Pilot study published in 2015.^92^	• *Access:* 26.3% of patients registered with diabetes were screened in the year before the intervention compared to 39.0% the year after the service was initiated.^92^
**Elderly healthcare voucher scheme (Hong Kong)**^93^	• *Service delivery:* National service that subsidises cost of private primary care for seniors aged 65 years or above. • *Medical technologies:* Eye care consultations, refractive error correction. • *Health workforce:* Vouchers are redeemable with private primary health care providers, including optometrists. • *Health financing:* Government funded. Electronic voucher provides HK$2000/∼US$257 every two years and unspent funds can accumulate up to HK$8000/∼US$1026. Spending on optometry services is capped at $2000 every two years • *Leadership & governance:* Co-ordinated by the Department of Health. • *Health information:* Not identified.	• *Access*: The number of older adults who used vouchers increased from 28% in 2009 to 94% in 2018.^38^ • *Integration:* The scheme does not encourage the use of private services for the management of chronic conditions as intended, (e.g. 54% of voucher claims for acute care vs 13% for chronic care in 2017) but does provide alternative health care options for older people. Participation by healthcare providers increased between 2014 to 2017, and optometrists had the highest participation rate (78% in 2017). Future policies may allow voucher use within the public sector.^38^ • *People-centredness*: The scheme applies a “money follows the patient” concept, allowing patients to choose their own private primary healthcare providers. 95% of older adults interviewed in 2016 agreed that the scheme was convenient to use, and 72% considered the coverage of healthcare services sufficient.^38^ • *Financial protection*: Vouchers cannot be used solely for the purchase of products (e.g. spectacles). However, healthcare practitioners can exploit patients to spend vouchers on unnecessary products (e.g. expensive frames and lenses) to maximise profits. Spending on optometry services is disproportionately high.^38^
**Hospital Authority's Risk Assessment and Management Programme (Hong Kong)**^94^	• *Service delivery:* Annual retinopathy screening for people with diabetes. • *Medical technologies:* Habitual and pinhole visual acuity testing. Non-stereoscopic digital colour retinal fundus photography of each eye (with dilation). • *Health workforce:* Screening is conducted in general outpatient clinics by trained graders, optometrists, or ophthalmologists. Patients with detected disease are referred to specialist ophthalmology clinics of the Hospital Authority. • *Health financing:* Government funded. Co-payment (HK$50 / ∼$US6) required per assessment, although fee waiver mechanism applies for eligible people. • *Leadership & governance*: Risk Assessment and Management Programme (RAMP) coordinated by the Hospital Authority. • *Health information*: Graders undergo structured training programme, and quality assurance assessment. No audits identified.	• *Financial protection*: Providing screening services free of charge improves uptake compared to screening with a small co-payment.^22^

#### What is offered and who for?

**Figure 1:**
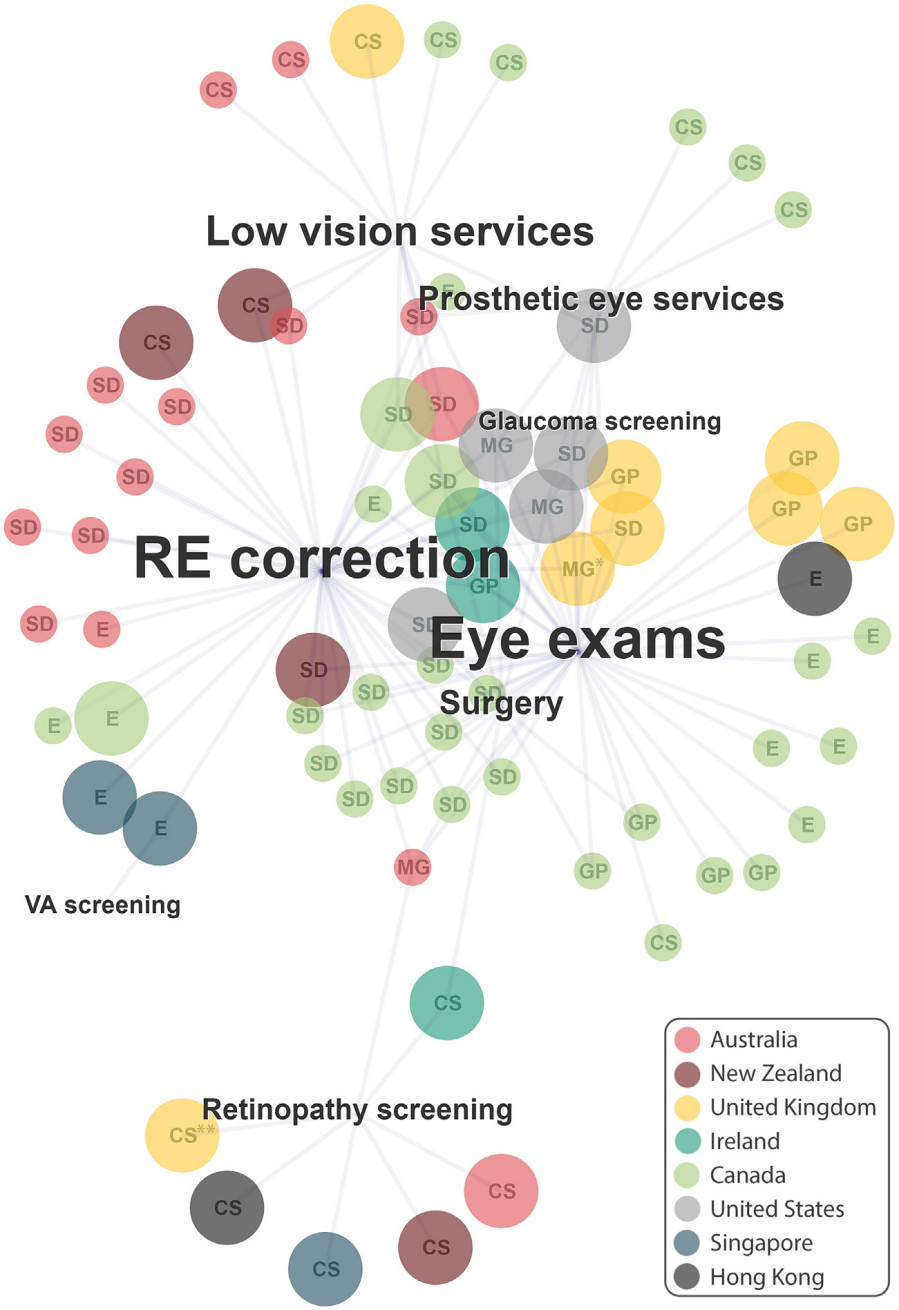
Network diagramme illustrating the types of services offered by the included 76 eye care programmes across 11 high-income countries/territories. Each programme is illustrated by a single circle: colour indicates the country that offers the programme (see key); size indicates the programme scope (small circles=state or provincial, large circles=national programmes; letters indicate eligible population: E=Elderly, SD=socially disadvantaged, CS=condition specific, GP=general population, MG=multiple groups). Each programme offers one or more of eight services, illustrated by text labels: size of the text represents the relative proportion of programmes offering that service. Services: Refractive error correction (RE correction; *n*=44), eye exams (*n=*42), low vision services (*n=*13), diabetic retinal screening (*n=*10), prosthetic eye services (*n=*8), surgery (*n=*8), glaucoma screening (*n=*3), visual acuity (VA) screening (*n=*1). *Indicates parallel services offered within England, Northern Ireland, and Wales; **Indicates parallel services offered within England, Northern Ireland, Scotland, and Wales.

The 76 eye care programmes offered a range of assessment and treatment services. Some ran isolated services, while others offered a combination of several services (Table 2, Figure 1). In general, programmes that targeted broad population groups (e.g. socially disadvantaged people) offered comprehensive services including eye exams, refractive error correction, and surgery (shown as the core services in the centre of Figure 1), and these were mostly staffed by clinicians. Programmes offering specific services (e.g. screening or rehabilitation) were less likely to provide general eye care and were administered by technical workers (shown at the edges of Figure 1).

All countries had at least one programme offering visual acuity assessment (Singapore) or eye examinations (all other countries); New Zealand was the only country without a programme that provided this care without requiring out-of-pocket payments from groups least able to pay. Of the 43 programmes offering eye examinations, 27 also included refractive error correction, and an additional 15 subsidy programmes offering refractive error correction were identified (ten of which were across eight states/territories of Australia). Fourteen screening services were identified: national-level diabetic retinal screening programmes in nine countries, three glaucoma screening services in the USA packaged within comprehensive eye care programmes, one programme in Australia that encouraged service providers to enrol people with diabetes into a reminder system to promote access to retinal screening, and one visual acuity screening service in Singapore. Two of the screening services included follow-up care within the service itself (i.e. Ireland's Diabetic RetinaScreen, and Singapore's Project Silver Screen that included some funding for refractive error correction, Table 3). Nineteen rehabilitation services were identified from five countries (New Zealand, Australia, Wales, Canada, and the USA); thirteen of these provided low vision aids (e.g. Welsh Low Vision Service, Table 3) and eight provided prosthetic eye services.

Populations eligible for each programme varied, and some programmes targeted multiple population groups (Table 2, Figure 1). Diabetic retinal screening, glaucoma screening, and low vision or prosthetic eye services were available specifically for people with these eye conditions or needs. Eighteen services targeted older people (thirteen of these exclusively), including one screening programme, twelve general eye care programmes offering eye examinations, and five subsidies for refractive error correction. The population group most commonly eligible for the services we identified were those experiencing social disadvantage, targeted by 33 services (offering eye examinations and/or refractive error correction) including General Ophthalmic Services in England, Northern Ireland and Wales, and the Victorian Aboriginal Spectacle Subsidy Scheme in Australia (Table 3). Ten services offering eye examinations were available to the general population (mostly in the UK and Canada), including the General Ophthalmic Services Scotland (Table 3).

#### Who provides the service and where?

The 76 eye care programmes were administered by a range of service providers, and some programmes by multiple provider types and different locations (Table 2). Clinicians working in optometry (e.g. opticians or optometrists) or ophthalmology (e.g. ophthalmic practitioners or ophthalmologists) were the most common (55 and 37 services respectively). Eight (of nine) services provided by technicians or nurses, and two (of four) services provided by GPs (e.g. Singapore's Primary Care Networks, Table 3) were screening services. The six services operated by technical workers (e.g. ocularists and low vision assessors) were low vision and prosthetic eye services. The location of the service was described for about half of the identified programmes (*n=*39). These services were provided at healthcare centres (including public clinics, private practices, or hospitals) and/or community locations not conventionally dedicated for healthcare (e.g. mobile clinics or community centres).

#### How is it funded?

Seventy-four of the 76 identified services were publicly funded (e.g. Medicare in Australia and the USA, the UK's National Health Service), while two services reported corporate funding in addition to public funding (Australia's KeepSight, and Singapore's Project SilverScreen).

#### What does the user pay?

Twenty services required no out-of-pocket payment by the user: nine of these provided eye examinations, six were national diabetic retinal screening services, and four were refractive error subsidies or low vision services. Most services required the user to pay an additional fee or co-payment for basic service. Payment for services were made directly to the service provider or by reimbursement to the service user, although we also identified eight services where monetary vouchers were provided to the service user, (including Hong Kong's Elderly Healthcare Voucher Scheme, Table 3). The eye examination service offered in New Zealand differed from the service offered within other countries, as it was provided as a financial loan for eye care.

#### Who is accountable for maintaining service quality?

Limited information was available describing the governance structure and health reporting within the included services. We identified fourteen services that reported how the service was governed, eight services that acknowledged communication with stakeholders, and ten services with published health monitoring standards. We also identified eighteen published audits of service performance (from 11 services) (outlined below in Question 2).

### Question 2: Availability of evidence

**Figure 2:**
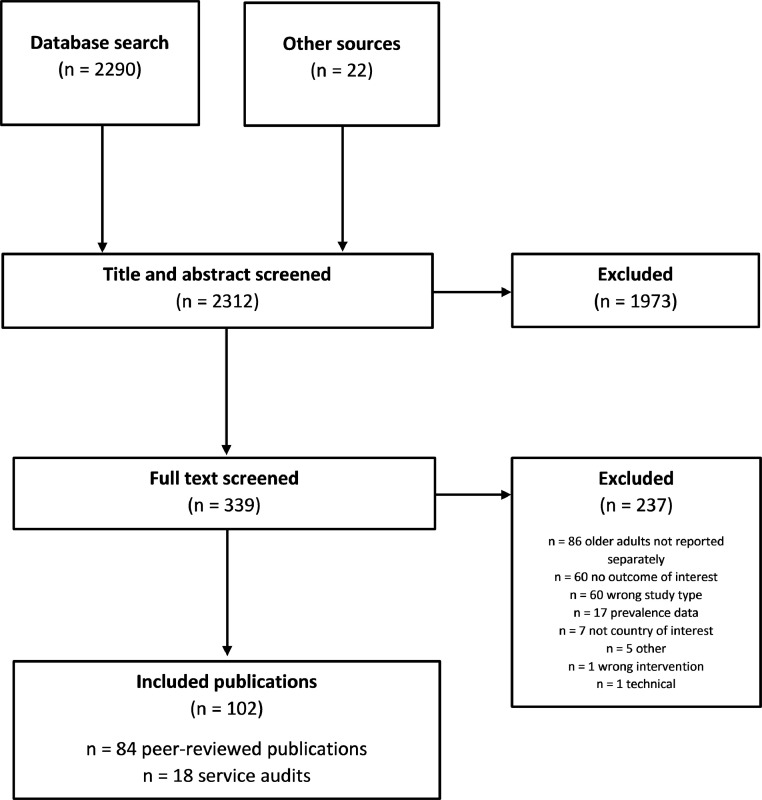
PRISMA flow diagram summarising the screening and selection of evidence to answer Question 2 of the review.

Of the 2,312 publications retrieved from our search, we examined the full-text of 339 and ultimately included 102 in our synthesis: 84 peer-reviewed studies and 18 service audits identified from Question 1 (Figure 2). Most publications reported findings from cross-sectional or cohort studies (*n=*73) and described eye care services in the UK (*n=*39), USA (*n=*21), or Australia (*n=*15); almost twice as many were published in the second half (*n=*68) compared to the first half (*n=*34) of the decade. Forty-two publications reported on diabetic retinopathy, 18 on glaucoma services, and 32 reported general eye care services not pertaining to any particular condition (Table 4).

**Table 4:** Characteristics of included studies reporting Universal Health Coverage outcomes from eye care services within the included countries.

Study Characteristics			n (*N=*102)	%
**Country**	UK		39	38.2
	USA		21	20.6
	Australia		15	14.7
	Canada		5	4.9
	Ireland		6	5.9
	Singapore		5	4.9
	Hong Kong		4	3.9
	International		5	4.9
	New Zealand		1	1.0
	New Zealand /Australia		1	1.0
**Study design**	Cross-sectional or cohort		73	71.6
	Quasi-experiment		15	14.7
	Meta-analysis		5	4.9
	Mixed methods		4	3.9
	Randomised Controlled Trial (RCT)		2	2.0
	Survey		2	2.0
	Open controlled trial		1	1.0
**Year of publication**	2010-2014		34	33.3
	2015-2020		68	66.7
**Eye condition**	Diabetic retinopathy		42	41.2
	General		32	31.4
	Glaucoma		18	17.6
	Cataract		6	5.9
	Uncorrected refractive error		3	2.9
	Age-related macular degeneration		1	1.0
**Outcome**^a^	Change in vision impairment		7	6.0
	Access		70	68.6
	Equity		47	46.1
	Financial protection		5	4.9
	Quality	Integration	39	38.2
		Efficiency	28	27.5
		Timeliness	18	17.6
		Safety	11	10.8
		People-centredness	10	9.8

a As each publication can report multiple outcomes, the total number of publications sums to >102.

**Figure 3:**
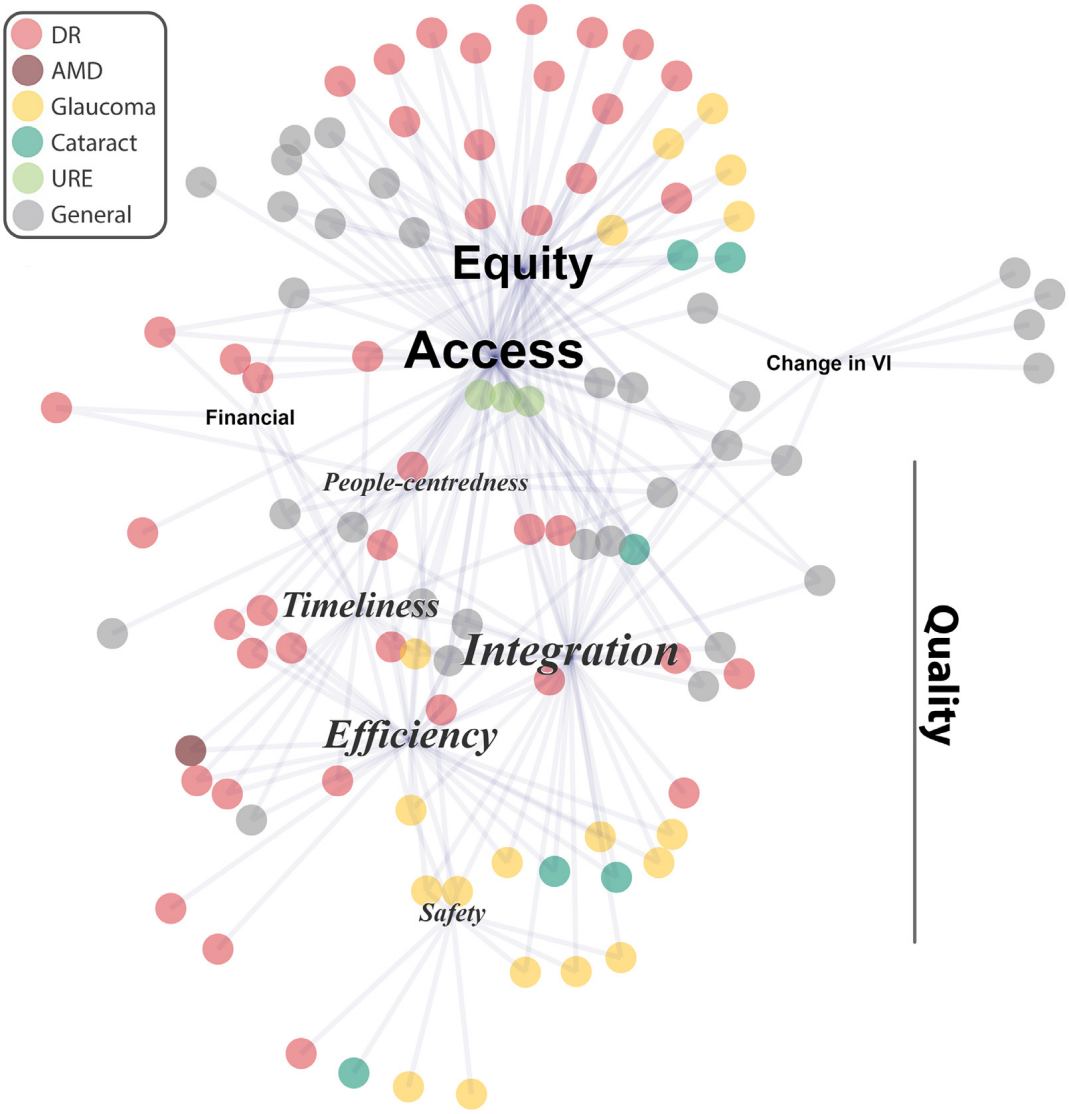
Network diagramme illustrating the Universal Health Coverage dimensions of eye health services reported by the included publications (*n=*102). Each publication is illustrated by a single circle: colour indicates the vision condition that the published evidence described (see key). Each publication reported one or more UHC dimensions, illustrated by text labels, whereby the size of the text illustrates the relative proportion of publications reporting that dimension. UHC dimensions: access (*n=*70), equity (*n=*47), quality (integration (*n=*39), efficiency (*n=*28), timeliness (*n=*18), safety (*n=*11), people-centredness (*n=*10), change in vision impairment (*n=*7), financial protection (*n=*5); Quality dimensions are shown in italics; VI=vision impairment, DR = diabetic retinopathy, AMD=age-related macular degeneration; URE=uncorrected refractive error.

Most included publications reported more than one outcome (i.e. (i) change in vision impairment, and/or (ii) any of the UHC dimensions: media*n=*2 per publication, interquartile range (IQR) 2-3; Table 4; Figure 3). The identified evidence is summarised in brief below across each outcome (and detailed in Supplementary Table 3), and the evidence for the specific programmes identified in Question 1 (detailed in Table 3) is highlighted.

#### Reduction in vision impairment

The seven publications reporting changes in vision impairment described general eye care services (Figure 3). These included three systematic reviews each concluding that there is insufficient evidence to support community vision screening for vision impairment in older people.^13^, ^14^, ^15^, ^16^

#### Universal health coverage for eye health

Access to services was the most reported UHC dimension (*n=*70 publications; Table 4). Audits of UK's NHS-funded eye care services illustrated increased use of these services over time, and that people aged ≥65 years received the largest proportion of funded eye examinations (Table 3).^17^, ^18^, ^19^, ^20^ Equity was also a frequently reported UHC dimension (*n=*47), with substantial overlap between publications describing both access and equity (*n=*45 publications; Figure 3). Scotland's universal eye care policy improved access to eye care services overall, but these improvements were less apparent in population groups with lower income or lower education, suggesting that the policy may have widened socioeconomic inequities (Table 3).^21^ Thirty-one publications identified the socioeconomic factors associated with poorer access to eye screening or follow-up; under-served population groups included those with lower levels of education or income, non-Caucasian ethnicities, those without health insurance, and people living in areas that are remote or with high area-level deprivation (Supplementary Table 3).

Financial protection was reported infrequently (*n=*5), and the included evidence illustrated that even minimal costs present a barrier to accessing eye care. An RCT conducted within the Hong Kong Hospital Authority showed poorer access to diabetic retinal screening when a small co-payment was required compared to a service with no out-of-pocket costs,^22^ and an audit of New Zealand's low vision services illustrated that some vision aids are expensive, which may reduce their accessibility^23^ (Table 3).

Publications reporting quality dimensions were less connected to access, equity, or financial protection (Figure 3). Of the quality dimensions, integration (*n=*39) was commonly reported (Table 4). Eleven of these reported the integration (± safety dimension) of glaucoma eye care services^24^, ^25^, ^26^, ^27^, ^28^, ^29^, ^30^, ^31^, ^32^, ^33^, ^34^ in the UK that refined referrals or allowed shared management and post-operative care of low-risk patients by community optometrists. These services reduced the burden on ophthalmologists in hospital eye care services by minimising false-positive referrals and managing patients outside the hospital system. Nine publications illustrated how eye care services (including primary diabetic screening) can be integrated into primary care via general practice (Supplementary Table 3). Eight publications from Australia illustrated the importance of well-integrated, culturally safe eye care services for Indigenous people (Supplementary Table 3), such as the Victorian Aboriginal Spectacle Subsidy Scheme^35^, ^36^, ^37^ (Table 3). In Hong Kong, a scheme encouraging older people to seek primary health care from private rather than public providers has had mixed results (Table 3).^38^

The efficiency of eye care services was also a commonly reported quality dimension (*n=*28), which frequently overlapped with timeliness (*n=*18; 12 of which also reported an efficiency outcome; Table 4, Figure 3). Data from the national diabetic eye screening programmes in England,^39^ Scotland,^40^^,^^41^ and Ireland^42^, ^43^, ^44^ reported long wait times for screening and a high referral rate during the initial years of the programme, however this reduced within a few years as existing conditions were detected and treated. Eight publications described how teleophthalmology can improve the efficiency of diabetic retinopathy,^45^, ^46^, ^47^, ^48^, ^49^ age-related macular degeneration (AMD),^50^ general vision screening,^51^ or ophthalmology^52^ services (Supplementary Table 3). Safety (*n=*11) and people-centredness (*n=*10) were the least reported quality dimensions (Table 4; Supplementary Table 3).

## Discussion

This systematic scoping review includes a broad range of evidence describing the types of eye health services for older adults that are available within eleven high-income countries/territories. In addition to evidence for a reduction in population vision impairment, we sought evidence for the extent to which eye care services are addressing UHC dimensions. We aimed to inform a specific policy being considered in New Zealand,^6^ but our findings are relevant more generally as countries attempt to improve UHC for eye health.^1^ In general, we observed a disparity between what existing services offer (Question 1) and the research focus (Question 2): most services targeted underserved populations with general care, while research most commonly focused on diabetes services.

The initial idea proposed for improving eye health among older adults in New Zealand was visual acuity screening for everyone aged ≥65 years.^6^ We found no evidence to support vision screening without also providing adequate follow-up care. Indeed, we identified reviews by three separate groups that determined there was no or inconclusive evidence to support community vision screening for impaired vision in older people, including from Cochrane, and both the Canadian and US Preventative Task Force.^13^, ^14^, ^15^, ^16^ Singapore was the only country we identified with a vision screening programme specifically for older adults.^53^ Eye care policies from other countries favour more comprehensive eye care services. For example, Scotland replaced the NHS “sight test” with a comprehensive eye examination in 2006,^54^ and more recently expanded this so clinicians can perform additional follow-up tests.^19^ Beyond Scotland, the other included countries commonly provided a fully or partially subsidised eye examination, generally performed by an optometrist and available to a subset of the population most in need of subsidised services, which often included people aged ≥65 years. Many of the identified services also had integrated follow-up treatment, including subsidised refractive error correction. New Zealand was the only country included in this review without any subsidised eye examinations or refractive error correction for older adults.

The most common UHC dimensions reported in the literature were access and equity, followed by quality (particularly the quality dimensions of integration and efficiency); very few publications reported on financial protection. Our review provides some key considerations for decision-makers in pursuit of UHC for eye health in their country.

Achieving UHC for eye health among older adults will require decision-makers to incorporate equity into the design of services, including targeting the most underserved populations subgroups. Our included evidence commonly reported inequities in access to eye care for people aged ≥65 years particularly if they were of a non-Caucasian ethnicity, had low education or income, did not have health insurance, or lived in areas that were remote or with high deprivation. To address these disparities, we found evidence that eye care services operated by culturally safe service providers in a community setting rather than formal eye health care setting can reach more older people from minority or underserved groups,^55^ particularly in rural and remote locations.^49^ Another strategy could involve lower age thresholds for population groups with lower life expectancy to become eligible for programmes, such as Māori and Pacific people in New Zealand.^56^

Integration and the continuum of care across levels was another common theme in this review, evident in some but not all countries and featured in more than one-third of the literature we identified. Integration is currently a key priority in global eye health. In the World Report on Vision, WHO outlined the need for Integrated People-centred Eye Care (IPEC) to achieve UHC,^1^ and this was endorsed by all Member States via a World Health Assembly Resolution in 2020.^2^ Further, integration featured twice in the top 10 priorities following a recent Grand Challenges in global eye health prioritisation process, highlighting the need for better vertical integration between levels of eye health services, and horizontal integration with other health services.^57^ We found examples of both of these.

One horizontal integration example we identified was retinal screening for people with diabetes delivered via general practice, which increased retinal screening rates.^58^ Strategies like this that increase access to primary eye health services will increase detection of conditions requiring treatment which can lead to increased demand for tertiary care.^57^ We found examples of vertical integration strategies to mitigate this, including shared-care between optometrists and ophthalmologists in the UK for people with glaucoma^24^ and cataract.^59^ A common theme of studies reporting elements of service integration was the importance of good communication between providers and enabling technology—including teleophthalmology and secure electronic health information systems—to enhance efficiency and timeliness of the service.^57^

Refractive error correction is a condition that highlights the importance of integrated eye care services. Refractive error is the leading cause of vision impairment globally, including among older adults.^60^ The importance of its correction in the pursuit of UHC was confirmed by the World Health Assembly Resolution in 2021, where Member States signed up to ambitious targets to increase effective refractive error coverage (eREC).^61^ Of our included countries, Australia is the only one which has an estimate of eREC—in 2016 approximately 9 out of 10 non-Indigenous (eREC: 93.5%) and 8 out of 10 Indigenous Australians (eREC 82.2%) requiring refractive error correction had it and could see 6/12 (i.e. were not vision impaired).^62^ While this inequity between the two groups must be overcome, we believe the spectacle subsidies available in Australia make an important contribution to Australia currently having the highest eREC estimates, albeit among a small group of countries with data available.^4^ This can be confirmed when eREC estimates become available for other countries with spectacles subsidies, including the UK and Canada.

Comprehensive monitoring to assess effectiveness of the 76 programmes we identified in Question 1 was largely unavailable—we found reports on 11 of these services, the majority from service audits or other grey literature. While most of these reported positive outcomes, including increased use of services or improved efficiency over time, it is unclear whether these services are adequate to meet the needs of the population. Moreover, a lack of evidence within the included UHC dimensions may be a result of under-reporting, rather than a lack of adherence to eye care services within UHC principles. In pursuit of UHC for eye health, more and better evidence is required on what works, for whom, and in what circumstances—this can include better use of routinely collected information, as well as via stronger partnerships between researchers and decision-makers to answer policy-relevant questions. The scoping nature of our review and the heterogeneity of the included evidence precluded critical appraisal of the evidence we identified. As eye health researchers respond to the call made by WHO^1^ and the Lancet Commission^4^ to generate more UHC-aligned evidence in the coming years, more extensive synthesis, including critical appraisal will be possible.

Finally, we found very few studies reporting outcomes relating to financial protection and those that did highlighted the need for it rather than demonstrating ways in which providing it improved coverage of services. Out-of-pockets costs are a major barrier to eye health,^4^ and the urgent need to address this dearth of evidence on financial protection was recognised in the recent Grand Challenges in global eye health exercise.^57^

Our review must be considered in light of several limitations. First, we limited our search to English-speaking countries with health services comparable to New Zealand. While the range of programmes identified provide useful information for New Zealand and similar countries, there may be strategies to improve eye health in older adults in countries that are absent from this summary. Second, we accessed information from public-facing websites which meant the details we sought on service structure were not always available. To reduce missing information, where possible we verified our comprehensive search results with researchers familiar with the health system in that country. Third, due to the focus of our search on primary-level services for older adults, we may have missed publications that report on relevant services that are available at other levels and to a larger age range. Despite these limitations, we have found a broad range of relevant evidence that can be considered by countries wanting to improve access to eye care among older adults.

## Conclusion

In most of the high-income countries included in this review, eye care examinations are available for people aged ≥65 years for little or no out-of-pocket cost. New Zealand is a notable outlier, with no subsidised general eye health services for older adults. Future eye care policies in New Zealand or elsewhere should incorporate UHC in their design, by targeting services towards underserved population groups, and integrating eye health services within the current health system. This could be achieved via horizontal integration with other primary care services and vertical integration that allows shared-care between optometrists and tertiary care providers. Future research in this area could investigate how financial protection for the service user can encourage access to services.

## Contributors

The review was conceptualised by JR, JE, and LG. Supervision was provided by JR and JE, and project administration performed by LG. Grey literature searching on government websites was performed by LG with assistance from SM, LK, and BT. IG constructed the search. Screening of abstracts and full-text reports was performed by LG, JR, JB, BT, SM, and SW. Data extraction from included reports was completed by LG, and confirmed by JB, BT, SM, and SW. Data curation was performed by LG. Data synthesis was performed by LG, LH, and JR. Figures were created by LH. LG and JR drafted the manuscript, and feedback was provided by all authors.

## Declaration of interests

I/we declare no competing interests.

## Appendix Supplementary materials

Image, application 1
