# Supplementary material for: Primary eye health services for older adults as a component of universal health coverage: a scoping review of evidence from high income countries
Source: Lancet Reg Health West Pac. 2022 Aug 12;35:100560. doi: 10.1016/j.lanwpc.2022.100560 (PMC10326716; doi:10.1016/j.lanwpc.2022.100560)
Supplement: Supplementary file 1 [file mmc1.pdf]

Supplementary material for:

Primary eye health services for older adults as a component of universal health coverage: A scoping review of evidence from high income countries *Lancet Regional Health – Western Pacific* 2022

Supplementary Table 1: Population, health expenditure, and service coverage descriptors of the eleven countries/territories included in this review

Country	Population[1] (millions)	Health expenditure per capita[2] ($US)	UHC 3.8.1[3]* (index)
**New Zealand**	4.98	4211.05	87.15
**Australia**	25.37	5427.46	85.53
**United Kingdom**	66.83	4312.89	87.72
**Ireland**	4.93	5428.59	82.81
**Hong Kong**	7.51	-	-
**Singapore**	5.70	2632.71	85.87
**Canada**	37.60	5048.37	89.36
**USA**	328.33	10921.01	82.61

*WHO's UHC service coverage index summarises health service coverage across 14 indicators and is reported on a scale of 1-100.[3]

[1] The World Bank. Population, total | Data [Internet]. The World Bank Group. 2019 [cited 2020 Sep 15]. Available from: https://data.worldbank.org/indicator/SP.POP.TOTL
[2] The World Bank Group. Current health expenditure per capita (current US$) | Data [Internet]. [cited 2022 Jul 12]. Available from: https://data.worldbank.org/indicator/SH.XPD.CHEX.PC.CD?end=2019&most_recent_value_desc=true&start=2019&view=map
[3] World Health Organization. UHC service coverage index (3.8.1) [Internet]. [cited 2022 Jul 11]. Available from: https://www.who.int/data/gho/indicator-metadata-registry/imr-details/4834

Supplementary Table 2: Search strategy used to identify evidence for effectiveness of eye care services within eleven high-income countries (Question 2).

Medline
1. Mass Screening/
2. Vision Screening/
3. Vision Tests/
4. Visual Field Tests/
5. screen$.tw.
6. Preventive Health Services/
7. Health Promotion/
8. Diagnostic Services/
9. Early Diagnosis/
10. Primary Health Care/
11. Community Health Services/
12. (communit$ adj2 eye adj2 (health or care or service$ or provision)).tw.
13. or/1-12
14. Humans/
15. exp Adult/
16. (adult or adults).tw.
17. (geriatric$ or elderly or senior$).tw.
18. people.tw.
19. or/14-18
20. 13 and 19
21. Geriatric Assessment/
22. Health Services for the Aged/
23. or/21-22
24. 20 or 23
25. exp Cataract/
26. cataract$.tw.
27. Retinal Degeneration/ or Macular Degeneration/ or Wet Macular Degeneration/
28. ((macul$ or retina$) adj2 degener$).tw.
29. exp Glaucoma/
30. (glaucoma$ or ocular hypertension).tw.
31. Diabetic Retinopathy/
32. ((diabet$ or proliferat$) adj3 retinopath$).tw.
33. (diabet$ adj3 (eye$ or vision or visual$ or sight$)).tw.
34. (retinopath$ adj3 (eye$ or vision or visual$ or sight$)).tw.
35. (dilated adj2 fundus).tw.
36. (retinal adj2 exam$).tw.
37. exp Refractive Errors/
38. (myopia or myopic or myopes or hyperop$ or hypermetrop$ or presbyop$).tw.
39. (refractive adj1 error$).tw.
40. Eyeglasses/
41. (spectacle or spectacles).tw.
42. (eyeglasses or eye glasses).tw.
43. Vision, Low/
44. ((low$ or impair$ or partial$ or loss$ or limit$) adj3 (vision or visual$ or sight$)).tw.
45. exp Visual Acuity/
46. (eye adj2 (health or disease$ or problem$)).tw.
47. (ocular adj1 condition$).tw.
48. or/25-47
49. 24 and 48
50. exp Australia/
51. Australia.ti,ab.
52. Brunei/
53. Brunei.ti,ab.
54. French Polynesia.ti,ab.
55. Guam/
56. Guam.ti,ab.
57. Hong Kong/
58. Hong Kong.ti,ab.
59. exp Japan/
60. Japan.ti,ab.
61. Korea/ or "Republic of Korea"/
62. ((Korea or South Korea or (Republic adj1 Korea)) not (North Korea or (Democratic adj2 Korea))).ti,ab.
63. Macau/
64. (Macau or Macao).ti,ab.
65. New Caledonia/
66. New Caledonia.ti,ab.
67. New Zealand/
68. New Zealand.ti,ab.
69. Northern Mariana Islands.ti,ab.
70. Palau/
71. (Palau or Paulau).ti,ab.
72. Singapore/
73. (Singapore or Straits Settlements).ti,ab.
74. Taiwan/
75. Taiwan.ti,ab.
76. or/50-75 [HIGH INCOME COUNTRIES IN EAST ASIA AND THE PACIFIC]
77. Bermuda/
78. Bermuda.ti,ab.
79. exp Canada/
80. Canada.ti,ab.
81. exp United States/
82. (United States or (United States adj2 America) or USA).ti,ab.
83. or/77-82 [HIGH INCOME COUNTRIES IN NORTH AMERICA]
84. Andorra/
85. Andorra.ti,ab.
86. Austria/
87. Austria.ti,ab.
88. Baltic States/
89. (Baltic adj1 (state? or countr* or region)).ti,a
90. Belgium/
91. Belgium.ti,ab.
92. Benelux.ti,ab.
93. Croatia/
94. Croatia.ti,ab.
95. Czech Republic/
96. (Czech Republic or Czechia or Czechoslovakia).ti,ab.
97. Cyprus/
98. Cyprus.ti,ab.
99. Denmark/
100. Denmark.ti,ab.
101. Estonia/
102. Estonia.ti,ab.
103. Faroe Islands.ti,ab.
104. Finland/
105. Finland.ti,ab.
106. exp France/
107. (France or French Republic).ti,ab.
108. exp Germany/

109. Germany.ti,ab.
110. Gibraltar/
111. Gibraltar.ti,ab.
112. Greece/
113. (Greece or Hellenic Republic).ti,ab.
114. Greenland/
115. Greenland.ti,ab.
116. Hungary/
117. Hungary.ti,ab.
118. Iceland/
119. Iceland.ti,ab.
120. Ireland/
121. ((Ireland or Eire) not Northern Ireland).ti,ab.
122. exp Italy/
123. (Italy or Italian Republic).ti,ab.
124. Latvia/
125. Latvia.ti,ab.
126. Liechtenstein/
127. Liechtenstein.ti,ab.
128. Lithuania/
129. Lithuania.ti,ab.
130. Luxembourg/
131. Luxembourg.ti,ab.
132. Malta/
133. Malta.ti,ab.
134. Mediterranean Region/
135. Mediterranean Islands/
136. Monaco/
137. Monaco.ti,ab.
138. Netherlands/
139. (Netherlands or Holland).ti,ab.
140. exp Norway/
141. Norway.ti,ab.
142. Poland/
143. Poland.ti,ab.
144. Portugal/
145. (Portugal or Portuguese Republic).ti,ab.
146. San Marino/
147. San Marino.ti,ab.
148. "Scandinavian and Nordic Countries"/
149. (((Scandinavian or Nordic) adj1 (countr* or state? or region)) or Scandinavia).ti,ab.
150. Slovakia/
151. (Slovakia or Slovak Republic).ti,ab.
152. Slovenia/
153. Slovenia.ti,ab.
154. Spain/
155. (Spain or Balearic Islands or Canary Islands).ti,ab
156. Sweden/
157. Sweden.ti,ab.
158. Switzerland/
159. (Switzerland or Swiss Confederation).ti,ab.
160. exp United Kingdom/
161. (United Kingdom or UK or Great Britain or GBR or Northern Ireland or Scotland or Channel Islands or Isle of Man or (Wales not New South Wales) or (England not New England)).ti,ab.
162. or/84-161 [HIGH INCOME COUNTRIES IN EUROPE AND CENTRAL ASIA]
163. (Queensland or New South Wales or South Australia or Tasmania or Victoria or Western Australia or Australian Capital Territory or Northern Territory).tw.

164. (Ontario or Quebec or Nova Scotia or New Brunswick or Manitoba or British Columbia or Prince Edward Island or Saskatchewan or Alberta or Newfoundland or Northwest territories or Yukon or Nunavut).tw.
165. (Alabama or Alaska or Arizona or Arkansas or California or Colorado or Connecticut or Delaware or Florida or Georgia or Hawaii or Idaho or Illinois or Indiana or Iowa or Kansas or Kentucky or Louisiana or Maine or Maryland or Massachusetts or Michigan or Minnesota or Mississippi or Missouri or Montana or Nebraska or Nevada or New Hampshire or New Jersey or New Mexico or New York or North Carolina or North Dakota or Ohio or Oklahoma or Oregon or Pennsylvania or Rhode Island or South Carolina or South Dakota or Tennessee or Texas or Utah or Vermont or Virginia or Washington or West Virginia or Wisconsin or Wyoming).tw.
166. (Alaska or Hawaii or Puerto Rico or Guam or Virgin Islands or Northern Mariana Islands).tw.
167. or/163-166
168. 76 or 83 or 162 or 167
169. 49 and 168
170. exp Infant/
171. exp Child/
172. exp Pediatrics/
173. (boy$ or girl$ or child$ or minor$).tw.
174. (adolescen$ or juvenile$ or teen or teens or teenage$ or youth or youths).tw.
175. ((primary or elementary or high or secondary) adj1 school$).tw. 176. (schoolchild$ or schoolboy$ or schoolgirl$ or schoolage or highschool$).tw.
177. (paediatric$ or pediatric$).tw.
178. or/170-177
179. 169 not 178
180. limit 179 to english language
181. limit 180 to yr="2010 -Current"
182. China/
183. India/
184. (China or Chinese or India or Indian).ti.
185. (screened adj5 independ$).tw.
186. (cell or cells or assay or genotyp$ or novel).ti.
187. or/182-186
188. 181 not 187
189. limit 188 to (comment or editorial or letter)
190. 188 not 189

Embase
1. mass screening/
2. vision test/
3. perimetry/
4. screen$.tw.
5. health promotion/
6. preventive health service/
7. early diagnosis/
8. primary health care/
9. community care/
10. (communit$ adj2 eye adj2 (health or care or service$ or provision)).tw.

11. or/1-10
12. human/
13. exp adults/
14. (adult or adults).tw.
15. very elderly/
16. (geriatric$ or elderly or senior$).tw.
17. people.tw.
18. or/12-17
19. 11 and 18
20. geriatric assessment/
21. elderly care/
22. or/20-21
23. 19 or 22
24. cataract/
25. cataract$.tw.
26. retina macula age related degeneration/ or age related macular degeneration/ or retina macula degeneration/ or macular degeneration/
27. ((macul$ or retina$) adj2 degener$).tw.
28. exp glaucoma/
29. (glaucoma$ or ocular hypertension).tw.
30. diabetic retinopathy/
31. ((diabet$ or proliferat$) adj3 retinopath$).tw.
32. (diabet$ adj3 (eye$ or vision or visual$ or sight$)).tw.
33. (retinopath$ adj3 (eye$ or vision or visual$ or sight$)).tw.
34. (dilated adj2 fundus).tw.
35. (retinal adj2 exam$).tw.
36. refraction error/
37. high myopia/ or hypermetropia/ or myopia/ or presbyopia/
38. (myopia or myopic or myopes or hyperop$ or hypermetrop$ or presbyop$).tw.
39. (refractive adj1 error$).tw.
40. spectacles/
41. (spectacle or spectacles).tw.
42. (eyeglasses or eye glasses).tw.
43. visual impairment/ or low vision/
44. ((low$ or impair$ or partial$ or loss$ or limit$) adj3 (vision or visual$ or sight$)).tw.
45. visual acuity/
46. (eye adj2 (health or disease$ or problem$)).tw.
47. (ocular adj1 condition$).tw.
48. or/24-47
49. exp "Australia and New Zealand"/
50. Australia.ti,ab.
51. Brunei Darussalam/
52. Brunei.ti,ab.
53. french polynesia/
54. French Polynesia.ti,ab.
55. Guam/
56. Guam.ti,ab.
57. Hong Kong/
58. Hong Kong.ti,ab.
59. Japan/
60. Japan.ti,ab.
61. korea/ or south korea/
62. ((Korea or South Korea or (Republic adj1 Korea)) not (North Korea or (Democratic adj2 Korea))).ti,ab.
63. Macao/
64. (Macau or Macao).ti,ab.
65. New Caledonia/
66. New Caledonia.ti,ab.
67. New Zealand.ti,ab.
68. northern mariana islands/
69. Northern Mariana Islands.ti,ab.
70. Palau/
71. (Palau or Paulau).ti,ab.
72. Singapore/
73. (Singapore or Straits Settlements).ti,ab.
74. Taiwan/
75. Taiwan.ti,ab.
76. or/49-75 [HIGH INCOME COUNTRIES IN EAST ASIA AND THE PACIFIC]
77. Bermuda/
78. Bermuda.ti,ab.
79. exp Canada/
80. Canada.ti,ab.
81. exp United States/
82. (United States or (United States adj2 America) or USA).ti,ab.
83. or/77-82 [HIGH INCOME COUNTRIES IN NORTH AMERICA]
84. Andorra/
85. Andorra.ti,ab.
86. Austria/
87. Austria.ti,ab.
88. Baltic States/
89. (Baltic adj1 (state? or countr* or region)).ti,ab.
90. exp Belgium/
91. Belgium.ti,ab.
92. benelux/
93. Benelux.ti,ab.
94. Croatia/
95. Croatia.ti,ab.
96. Czech Republic/
97. Czechoslovakia/
98. (Czech Republic or Czechia or Czechoslovakia).ti,ab.
99. Cyprus/
100. Cyprus.ti,ab. 101. Denmark/
102. Denmark.ti,ab.
103. Estonia/
104. Estonia.ti,ab.
105. faroe islands/
106. Faroe Islands.ti,ab.
107. exp Finland/
108. Finland.ti,ab.
109. exp France/
110. (France or French Republic).ti,ab.
111. exp Germany/
112. Germany.ti,ab.
113. Gibraltar/
114. Gibraltar.ti,ab.
115. Greece/
116. (Greece or Hellenic Republic).ti,ab.
117. Greenland/
118. Greenland.ti,ab.
119. Hungary/
120. Hungary.ti,ab.
121. Iceland/
122. Iceland.ti,ab.
123. Ireland/
124. ((Ireland or Eire) not Northern Ireland).ti,ab.
125. exp Italy/

126. (Italy or Italian Republic).ti,ab.
127. Latvia/
128. Latvia.ti,ab.
129. Liechtenstein/
130. Liechtenstein.ti,ab.
131. Lithuania/
132. Lithuania.ti,ab.
133. Luxembourg/
134. Luxembourg.ti,ab.
135. Malta/
136. Malta.ti,ab.
137. monaco/
138. Monaco.ti,ab.
139. Netherlands/
140. (Netherlands or Holland).ti,ab.
141. exp Norway/
142. Norway.ti,ab.
143. Poland/
144. Poland.ti,ab.
145. exp Portugal/
146. (Portugal or Portuguese Republic).ti,ab.
147. San Marino/
148. San Marino.ti,ab.
149. Scandinavia/
150. (((Scandinavian or Nordic) adj1 (countr* or state? or region)) or Scandinavia).ti,ab.
151. Slovakia/
152. (Slovakia or Slovak Republic).ti,ab.
153. Slovenia/
154. Slovenia.ti,ab.
155. exp Spain/
156. (Spain or Balearic Islands or Canary Islands).ti,ab.
157. Sweden/
158. Sweden.ti,ab.
159. Switzerland/
160. (Switzerland or Swiss Confederation).ti,ab.
161. exp United Kingdom/ or channel islands/ or "isle of man"/
162. (United Kingdom or UK or Great Britain or GBR or Northern Ireland or Scotland or Channel Islands or Isle of Man or (Wales not New South Wales) or (England not New England)).ti,ab.
163. or/84-162 [HIGH INCOME COUNTRIES IN EUROPE AND CENTRAL ASIA]
164. (Queensland or New South Wales or South Australia or Tasmania or Victoria or Western Australia or Australian Capital Territory or Northern Territory).tw.

165. (Ontario or Quebec or Nova Scotia or New Brunswick or Manitoba or British Columbia or Prince Edward Island or Saskatchewan or Alberta or Newfoundland or Northwest territories or Yukon or Nunavut).tw.
166. (Alabama or Alaska or Arizona or Arkansas or California or Colorado or Connecticut or Delaware or Florida or Georgia or Hawaii or Idaho or Illinois or Indiana or Iowa or Kansas or Kentucky or Louisiana or Maine or Maryland or Massachusetts or Michigan or Minnesota or Mississippi or Missouri or Montana or Nebraska or Nevada or New Hampshire or New Jersey or New Mexico or New York or North Carolina or North Dakota or Ohio or Oklahoma or Oregon or Pennsylvania or Rhode Island or South Carolina or South Dakota or Tennessee or Texas or Utah or Vermont or Virginia or Washington or West Virginia or Wisconsin or Wyoming).tw.
167. (Alaska or Hawaii or Puerto Rico or Guam or Virgin Islands or Northern Mariana Islands).tw.
168. or/164-167
169. 76 or 83 or 163 or 168
170. 23 and 48
171. 169 and 170
172. exp child/
173. exp infant/
174. exp adolescent/
175. exp pediatrics/
176. (boy$ or girl$ or child$ or minor$).tw.
177. (adolescen$ or juvenile$ or teen or teens or teenage$ or youth or youths).tw.
178. (paediatric$ or pediatric$).tw.
179. ((primary or elementary or high or secondary) adj1 school$).tw.
180. (schoolchild$ or schoolboy$ or schoolgirl$ or schoolage or highschool$).tw.
181. or/172-180
182. 171 not 181

183. limit 182 to english language
184. limit 183 to yr="2010 -Current"
185. China/
186. India/
187. (China or Chinese or India or Indian).ti.
188. (screened adj5 independ$).tw.
189. (cell or cells or assay or genotyp$ or novel).ti.
190. or/185-189
191. 184 not 190
192. limit 191 to conference abstract status
193. 191 not 192

Cochrane Library

#1 MeSH descriptor: [Mass Screening] this term only
#2 MeSH descriptor: [Vision Screening] this term only
#3 MeSH descriptor: [Vision Tests] this term only
#4 MeSH descriptor: [Visual Field Tests] this term only
#5 screen*
#6 MeSH descriptor: [Preventive Health Services] this term only
#7 MeSH descriptor: [Health Promotion] this term only
#8 MeSH descriptor: [Diagnostic Services] this term only
#9 MeSH descriptor: [Early Diagnosis] explode all trees
#10 MeSH descriptor: [Primary Health Care] this term only
#11 MeSH descriptor: [Community Health Services] this term only
#12 communit* near/2 eye near/2 (health or care or service* or provision)
#13 #1 or #2 or #3 or #4 or #5 or #6 or #7 or #8 or #9 or #10 or #11 or #12
#14 MeSH descriptor: [Humans] this term only

#15 MeSH descriptor: [Adult] explode all trees
#16 (adult or adults)
#17 (geriatric* or elderly or senior)
#18 people
#19 #14 or #15 or #16 or #17 or #18
#20 #13 and #19
#21 MeSH descriptor: [Geriatric Assessment] this term only
#22 MeSH descriptor: [Health Services for the Aged] this term only
#23 #21 or #22
#24 #20 or #23
#25 MeSH descriptor: [Cataract] explode all trees
#26 cataract*
#27 MeSH descriptor: [Retinal Degeneration] this term only
#28 MeSH descriptor: [Macular Degeneration] this term only
#29 MeSH descriptor: [Wet Macular Degeneration] this term only
#30 (macul* or retina*) near/2 degener*
#31 MeSH descriptor: [Glaucoma] explode all trees
#32 glaucoma* or ocular hypertension
#33 MeSH descriptor: [Diabetic Retinopathy] this term only
#34 (diabet* or proliferat*) near/3 retinopath*
#35 diabet* near/3 (eye* or vision or visual* or sight*)
#36 retinopath* near/3 (eye* or vision or visual* or sight*)
#37 dilated near/2 fundus
#38 retinal near/2 exam*
#39 MeSH descriptor: [Refractive Errors] explode all trees
#40 myopia or myopic or myopes or hyperop* or hypermetrop* or presbyop*
#41 refractive next error*
#42 MeSH descriptor: [Eyeglasses] this term only
#43 spectacle or spectacles
#44 eyeglasses or eye glasses
#45 MeSH descriptor: [Vision, Low] this term only
#46 (low* or impair* or partial* or loss* or limit*) near/3 (vision or visual* or sight*)
#47 MeSH descriptor: [Visual Acuity] explode all trees
#48 eye near/2 (health or disease* or problem*)
#49 ocular next condition*
#50 #25 or #26 or #27 or #28 or #29 or #30 or #31 or #32 or #33 or #34 or #35
#51 #36 or #37 or #38 or #39 or #40 or #41 or #42 or #43 or #44 or #45 or #46 or #47 or #48 or #49
#52 #50 or #51
#53 #24 and #50 in Trials
#54 MeSH descriptor: [Infant] explode all trees
#55 MeSH descriptor: [Child] explode all trees
#56 MeSH descriptor: [Pediatrics] explode all trees
#57 boy* or girl* or child* or minor*
#58 adolescen* or juvenile* or teen or teens or teenage* or youth or youths
#59 (primary or elementary or high or secondary) near/1 school*
#60 schoolchild* or schoolboy* or schoolgirl* or schoolage or highschool*
#61 paediatric* or pediatric*
#62 #54 or #55 or #56 or #57 or #58 or #59 or #60 or #61
#63 #53 not #62 with Publication Year from 2010 to 2020, in Trials
#64 MeSH descriptor: [Australia] explode all trees
#65 Australia
#66 MeSH descriptor: [New Zealand] this term only
#67 New Zealand
#68 MeSH descriptor: [Canada] explode all trees
#69 Canada
#70 MeSH descriptor: [United States] explode all trees
#71 United States or USA
#72 United States near/2 America
#73 MeSH descriptor: [Singapore] this term only
#74 Singapore
#75 Hong Kong
#76 MeSH descriptor: [United Kingdom] explode all trees
#77 United Kingdon or UK or Scotland or Wales or Northen Ireland
#78 MeSH descriptor: [Ireland] this term only
#79 Ireland
#80 Queensland or New South Wales or South Australia or Tasmania or Victoria or Western Australia or Australian Capital Territory or Northern Territory
#81 Ontario or Quebec or Nova Scotia or New Brunswick or Manitoba or British Columbia or Prince Edward Island or Saskatchewan or Alberta or Newfoundland or Northwest territories or Yukon or Nunavut
#82 Alabama or Alaska or Arizona or Arkansas or California or Colorado or Connecticut or Delaware or Florida or Georgia or Hawaii or Idaho or Illinois or Indiana or Iowa or Kansas or Kentucky or Louisiana or Maine or Maryland or Massachusetts or Michigan or Minnesota or Mississippi or Missouri or Montana or Nebraska or Nevada or New Hampshire or New Jersey or New Mexico or New York or North Carolina or North Dakota or Ohio or Oklahoma or Oregon or Pennsylvania or Rhode Island or South Carolina or South Dakota or Tennessee or Texas or Utah or Vermont or Virginia or Washington or West Virginia or Wisconsin or Wyoming
#83 Alaska or Hawaii or Puerto Rico or Guam or Virgin Islands or Northern Mariana Islands
#84 #64 or #65 or #66 or #67 or #68 or #69 or #70 or #71 or #72 or #73 or #74 or #75 or #76 or #77 or #78 or #79 or #80 or #81 or #82 or #83
#85 #63 and #84

CRD Database: DARE, NHS EED and HTA *Search 1*
Mesh term - Vision screening.
Search 2
Free text search - (Screen*) AND (Cataract* OR Macular Degeneration OR Glaucoma OR Diabetic Retinopathy OR Myopia OR Presbyopia OR Low Vision OR Visual Acuity OR glasses OR Spectacles) AND (Adult* OR Elderly OR Geriatric*) FROM 2010 TO 2020.

Supplementary Table 3: Summary of major findings reporting outcomes across Universal Health Coverage dimensions.

Outcome	# refs	Summary of major findings
**(i) Evidence that eye care services reduce vision impairment:**		
Reduction in vision impairment (7 publications)	*4*	***No evidence for general vision screening:***<br>• Insufficient evidence to support community vision screening for vision impairment in older people (three systematic reviews and one evidence report: Cochrane (high certainty evidence), Canadian Task Force on Preventative Health Care (low-moderate quality evidence), US Preventive Services Task Force (fair-good quality evidence).[1–4]
	1	• In an adult vision screening programme in the USA, no combination of screening question/s and distance visual acuity testing had a high sensitivity and specificity for detecting individuals who need eye care.[5]
	2	• Improved near visual acuity in people with low vision before and after accessing the Welsh Low Vision Service.[31,42]
**(ii) Evidence that eye care services address Universal Health Coverage dimensions for older adults:**		
Access (70 publications)	*48*	***Sub-optimal access to eye care services:***<br>• Attendance at diabetic retinal screening or follow-up services within the study period or at recommended frequencies ranged from 48.9% to 90.6% across the included screening programmes.[6–21]<br>• Access to general eye care services within the previous 12-24 months by people aged 40 or older ranged between 36.0-60.3% of participants in Canada, the UK, and USA.[22–24] Access to general eye care services at the recommended frequencies was 30.8% by Singaporeans with vision impairment[25], and 8.0-21.0% by women in the USA with self-reported eye disease[26].<br>• 57.0%[27,28] and 61.2%[29,30] of people with a glaucoma-related diagnosis from community vision screening in the USA attended their follow-up appointment.<br>• In Singapore, 83.3% of participants with diabetic retinopathy were undiagnosed,[31] 1.8% of glaucoma was undiagnosed,[32] and 68.6% of people with visually significant cataract were unaware of their cataract.[33]
	18	***Access differs across age groups***:<br>• Improved access with older age,[12,16,18,19,21,22,24–26,34,35] or reduced or delayed eye care in the ≥85 years age group[15,36] or in older Aboriginal and Torres Strait Islander people in Australia.[13]<br>• In the UK, people aged ≥65 years received the largest proportion of NHS-funded eye examinations.[37–40]
	1	***Interventions can improve access to eye care services:***<br>• ~12% increase in attendance at diabetic retinopathy screening with specific interventions aimed to encourage attendance (Cochrane systematic review of 66 randomised controlled trials).[41]
	4	***Improved access with funded services:***<br>• Service audits of the UK's NHS General Ophthalmic Services described a yearly increase in the number of claims in the ten years to 2018/19 across England[42], Northern Ireland,[37] Scotland,[43] and Wales.[40]<br>• Scotland's general ophthalmic services (which fund the general population)[38] had the highest attendance of all the devolved countries of the UK.[37]
Equity (47 publications)	*39*	***Socioeconomic factors associated with poorer access to eye care services:***<br>• Under-served population groups included those with lower levels of education or income,[10,12,19,20,22–24,44] non-Caucasian ethnicities,[8,10,20,21,44,45] those without health insurance,[11,12,20,26,44] and people living in areas that are remote[19,45] or with high area-level deprivation.[15,16,36,46]<br>• Scotland's universal eye examination policy improved access to eye care services overall, but these improvements were less apparent in population groups with lower income or lower education, suggesting that the policy may have widened socioeconomic inequities.[47]<br>• Low vision services in New Zealand[48] and Scotland,[49] cataract services in Canada,[50] and general ophthalmic services and diabetic eye screening programmes in Northern Ireland[37,51] and Wales[40,52] described inequitable access between ethnic groups or across geographical locations.

	8	***Well-integrated, culturally safe services in Australia encourage access to services:***<br>• The availability of an optometrist within Aboriginal Community Controlled Health Services had a greater impact on access to services than the equivalent private or hospital-based care.[53]<br>• Using a local health workforce in remote regions increased retinal screening rates for diabetes.[54]<br>• Within an urban area of Australia, re-designing an Indigenous cataract surgery service to provide more integrated and coordinated care increased the number of people completing surgery.[55]<br>The Victorian Aboriginal Spectacles Subsidy Scheme improved access to eye examinations for Indigenous people by subsidising refractive error correction, and a 2016 audit of the service described a positive impact on the Aboriginal community overall, despite variability in service provision and a lack of cultural awareness by some participating practices.[56–58]
Integration (39 publications)	18	***Eye care services that reduce the burden on public tertiary care:***<br>• Audits of Scotland's General Ophthalmic Services reported that 95% of patients were managed within community optometry,[38] and that the increase in referrals to hospital eye services over time was relatively small (13% over the last ten years).[38,39]<br>• In the UK, Glaucoma[59–69] and cataract[70,71] services that refine referral or allow shared management and post-operative care of low-risk patients are managed by community optometrists with specific training.<br>• A 2019/20 audit of the Northern Ireland Primary Eyecare Assessment and Referral Service reported that 66% of patients with urgent eye care needs were managed within the service and only 13% required urgent or routine referral to hospital eye care services[37].<br>• An audit of Ireland's eye care services recommended a revised model centred on community-based multidisciplinary care.[72]<br>• In Hong Kong, the Elderly Healthcare Voucher Scheme was developed to encourage older people to seek care from private rather than public providers. A 2018 audit[73] suggested that the service may not yet achieve this objective, but it does provide older people with more healthcare options.
	9	***Integration of eye care services with general practice:***<br>• People with diabetes in the USA were more likely to visit a primary care practitioner than an eye health provider,[44,74] suggesting that vision screening by general practitioners may reach most individuals, including those at high risk of retinopathy, and could therefore improve access for people who do not otherwise seek eye care.<br>• Observational studies from Australia and the USA reported that general practitioners do not typically prioritise eye care.[75–77]<br>• Different service models can successfully integrate eye care into general practice. For example, in Australia, an "over-75s health assessment" contained a visual acuity screening component conducted by general practitioners,[78] and diabetic retinopathy screening was successfully conducted in-house by general practitioners supported by ophthalmologists via telehealth.[79]<br>• Both Singapore's Primary Care Networks[80], and Ireland's Diabetes in General Practice[7] service coordinated retinal screening via general practices, but screening was conducted offsite (either by allied health workers or optometrists, respectively), and the results sent back to the GP for management.
Efficiency and timeliness (28/18 publications respectively)	11	***Managing referrals from primary care:***<br>• Data from the national diabetic retinopathy screening programmes in England,[81] Scotland,[82,83] and Ireland[84–86] reported long wait times for screening and a high referral rate during the initial years of the programme, however this reduced within a few years as existing disease was detected and treated.<br>• In Scotland, high rates of glaucoma referrals to tertiary care were reduced by adapting the referral guidelines and introducing local services to manage patients in the community.[61,68,69]
	8	***Teleophthalmology may improve efficiency and timeliness:***<br>• Within an open-controlled trial of Australian general practices, diabetic retinopathy screening rates were 33% following referral to local screening services compared to 100% using teleophthalmology, and higher follow-up rates in the teleophthalmology group.[79]<br>• An RCT of suspected AMD patients found no reduction in the referral to treatment time when comparing clinic-based screening by a specialist versus teleophthalmology screening with a basic assessment.[87] While treatment time was shorter in the clinic-based group for patients with recurring AMD, this had no effect on final visual outcomes, illustrating the safety of teleophthalmology for screening and monitoring of AMD.

		• In Scotland, a service that allowed electronic referrals from primary care to ophthalmology services together with examination of patients from digital images reported improved efficiencies such as reduced outpatient appointments and improved waiting times.[88] Therefore, these types of services may improve service efficiencies when used appropriately.
Safety (11 publications)	11	***Risk management by eye care providers:***<br>• Compliance by eye care providers to glaucoma assessment or management guidelines is less than optimal[89,90]<br>• Community management of glaucoma or cataract patients can operate with minimal levels of risk.[59,60,62,64,66,67]<br>• National data from Canada described how primary care practitioners with more diabetes-specific experience provided more appropriate diabetic eye care ,[91] and overall low complication rates after cataract surgery, with people aged 70-79 years requiring lower re-admission rates compared to those aged ≥80 years.[92]
People-centredness (10 publications)	10	***Illustrates a need for people-centred eye care services:***<br>• Patient satisfaction with the Welsh Low Vision Service.[93–95]<br>• A consultation with users and providers of Ireland's eye care services described service inefficiencies.[72]<br>• A review of the Hong Kong Elderly Healthcare Voucher Scheme illustrated its people-centred "money follows the patient" design, and positive perspectives of the service by its users.[73]<br>• An audit of the Victorian Aboriginal Spectacles Scheme suggested improvements in cultural awareness.[56–58]<br>• Other people-centred outcomes included the patient's choice of appointment times, and the time required to attend the service.[96,97]
Financial protection (5 publications)	2	***Low costs of vision screening:***<br>• The reported costs of attending diabetic eye care screening services in Wales were low.[96]<br>• For people living in rural USA, the travel costs could be reduced by accessing screening services via teleophthalmology.[97]
	3	***Illustrates a need for financial protection:***<br>• An RCT conducted within the Hong Kong Hospital Authority's Diabetic Retinopathy Screening Programme showed poorer access to diabetic retinopathy screening when a small co-payment was required compared to a service with no out of pocket costs.[98]<br>• An audit of New Zealand's low vision services illustrated that some vision aids are expensive, which may reduce their accessibility.[48]<br>• Hong Kong's Elderly Healthcare Voucher Scheme[73] reported disproportionately high spending on optometry services, with concerns that the scheme has encouraged spending on unnecessary ancillary products.

Supplementary Table 4: List of included eye care programmes for older adults within the eleven high-income countries (Question 1).

**Programme**
**New Zealand**
1. Diabetes Retinopathy Screening Services[99]
2. Equipment for people who are blind or have reduced vision[100]
3. Contact lens subsidy[101]
4. Work and Income benefit[102]
**Australia**
5. Medicare Australia[103]
6. Keep Sight[104]
7. Department of Veterans' Affairs optical services[105]
8. Queensland Spectacle Supply Scheme & the Indigenous Spectacle Supply Scheme[106]
9. New South Wales Spectacles Programme[107]
10. New South Wales Blindness and Low Vision Assistive Technology[108]
11. Australia Capital Territory Spectacles Subsidy Scheme[109]
12. Australia Capital Territory Seniors Spectacles Scheme[110]
13. Australia Capital Territory Low Vision Aids Scheme[111]
14. Victorian Eyecare Service[112]
15. Victorian Aboriginal Spectacles Subsidy Scheme[113]
16. Northern Territory Concession Scheme[114]
17. Glasses SA[115]
18. Western Australia Spectacle Subsidy Scheme[116]
19. Tasmania Visual Aids[117]
**Singapore**
20. Project Silver Screen (Functional Screening)[118]
21. Seniors' Mobility and Enabling Fund[119]
22. Primary Care Networks' Diabetic Eye Screening[120]
**Hong Kong**
23. Elderly healthcare voucher scheme[121]
24. Diabetic Retinopathy Screening Programme[122]
**Ireland**
25. Treatment Benefit Scheme[123]
26. Diabetic RetinaScreen[124]
27. Community Ophthalmic Services Scheme[125]
**United Kingdom**
28-31. National Health Service General Ophthalmic Services[38,126–128]
32. Covid-19 Urgent Eyecare Service England[129]
33. Northern Ireland Primary Eyecare Assessment and Referral Service (NI PEARS)[130]
34. Eye Health Examination Wales[131]
35. Low Vision Services Wales[132]
36-39. National Health Service Diabetic Eye Screening programme[133–136]
40. Support services for military and defence personnel[137]
**Canada**
41. Non-Insured Health Benefits Programme[138]
42. Veterans' Affairs Canada Vision Care Benefits[139]
43. Alberta Health Care Insurance Plan[140]
44. Alberta Dental and Optical Assistance for Seniors[141]
45. Alberta Adult Health Benefit[142]
46. British Columbia Medical Services Plan[143]
47. Ontario Health Insurance Plan[144]
48. Ontario Disability Support Program[145]
49. Ontario Assistive Devices Program[146]
50. Saskatchewan Universal Health Benefits[147]
51. Saskatchewan Supplementary Health Benefits[148]
52. Saskatchewan Seniors Income Plan[149]
53. Manitoba Health, Seniors and Active Living[150]
54. Manitoba Employment and Income Assistance[151]
55. Manitoba Seniors Eyeglass Program[152]
56. Manitoba Prosthetic Eye[153]
57. Nova Scotia Medical Services Insurance[154]
58. Nova Scotia Income Assistance[155]
59. Nova Scotia Disability Support Program[156]
60. Quebec Health Insurance Plan Optometric Services[157]

61. Quebec Health Insurance Plan Visual Devices Program[158]<br>62. Quebec Health Insurance Plan Ocular Prostheses Program[159]<br>63. Quebec Social Assistance and Social Solidarity[160]<br>64. New Brunswick Health Services Vision Program[161]<br>65. New Brunswick Ocular Prosthesis Program[162]<br>66. Prince Edward Island Optometry Service Program[163]<br>67. Prince Edward Island Social Assistance Program[164]<br>68. Newfoundland Labrador Income Support[165]<br>69. Northwest Territories Metis Health Benefits & Extended Health Benefits for Seniors Program[166]<br>70. Northwest Territories Eye Clinic Ophthalmology Services[167]<br>71. Yukon Pharmacare and Extended Health Care Benefits to Seniors[168]
**USA**
72. Medicare[169]<br>73. Medicaid (including the Basic Health Program)[170]<br>74. Veterans Affairs Vision Care[171]<br>75. TRICARE[172]<br>76. Civilian Health and Medical Program of the Department of Veterans Affairs (ChampVA)[173]

Supplementary Table 5: List of included studies reporting evidence of effectiveness within Universal Health Coverage dimensions (Question 2).

**General vision screening**	
1.	Clarke EL, Evans JR, Smeeth L. Community screening for visual impairment in older people. Cochrane Database Syst Rev. 2018 Feb 20;2018(2).
2.	Wilson BJ, Courage S, Bacchus M, Dickinson JA, Klarenbach S, Jaramillo Garcia A, et al. Screening for impaired vision in community-dwelling adults aged 65 years and older in primary care settings. Can Med Assoc J. 2018;190(19):E588–94.
3.	Chou R, Dana T, Bougatsos C, Grusing S, Blazina I. Screening for impaired visual acuity in older adults: Updated evidence report and systematic review for the US preventive services task force. J Am Med Assoc. 2016 Mar 1;315(9):915–33.
4.	Siu AL, Bibbins-Domingo K, Grossman DC, Baumann LC, Davidson KW, Ebell M, et al. Screening for impaired visual acuity in older adults: US preventive services task force recommendation statement. J Am Med Assoc. 2016 Mar 1;315(9):908–14.
5.	Lawrenson JG, Graham-Rowe E, Lorencatto F, Burr J, Bunce C, Francis JJ, et al. Interventions to increase attendance for diabetic retinopathy screening. Cochrane Database Syst Rev. 2018 Jan 15;2018(1):CD012054.
6.	Friedman DS, Cassard SD, Williams SK, Baldonado K, O'Brien RW, Gower EW. Outcomes of a vision screening program for underserved populations in the united states. Ophthalmic Epidemiol. 2013;20(4):201–11.
**General eye care**	
7.	Balegamire S, Aubin M-J, Curcio C-L, Alvarado B, Guerra RO, Ylli A, et al. Factors Associated With Visual Impairment and Eye Care Utilization: The International Mobility in Aging Study. J Aging Health. 2018;30(9):1369–88.
8.	Iliffe S, Kharicha K, Harari D, Swift C, Gillmann G, Stuck AE. Health risk appraisal in older people 6: factors associated with self-reported poor vision and uptake of eye tests in older people. BMC Fam Pract. 2013;14:130.
9.	Morales LS, Varma R, Paz SH, Lai MY, Mazhar K, Andersen RM, et al. Self-reported use of eye care among Latinos: the Los Angeles Latino Eye Study. Ophthalmology. 2010;117(2):207–15.
10.	Gupta P, Majithia S, Fenwick EK, Gan ATL, Tham Y-C, Poh S, et al. Rates and Determinants of Eyecare Utilization and Eyeglass Affordability Among Individuals With Visual Impairment in a Multi-Ethnic Population-Based Study in Singapore. Transl Vis Sci Technol [Internet]. 2020 Apr 18;9(5):11. Available from: https://tvst.arvojournals.org/article.aspx?articleid=2765019
11.	Centers for Disease Control and Prevention (CDC). Eye-care utilization among women aged > or =40 years with eye diseases--19 states, 2006-2008. Vol. 59, Morbidity and mortality weekly report. 2010.
12.	NHS Digital. General Ophthalmic Services Activity Statistics England, year ending 31 March 2020 - NHS Digital [Internet]. 2020 [cited 2020 Nov 29]. Available from: https://digital.nhs.uk/data-and-information/publications/statistical/general-ophthalmic-services-activity-statistics/england-year-ending-31-march-2020
13.	HSC Public Health Agency of Northern Ireland. General Ophthalmic Services Statistics for Northern Ireland, Annual Statistics 2019/20 [Internet]. FPS General Ophthalmic Services Statistics for 2019/20. 2020 [cited 2020 Nov 29]. Available from: http://www.hscbusiness.hscni.net/pdf/General Ophthalmic Statistics Publication 2019-20.pdf
14.	Information Services Division Scotland. General Ophthalmic Services Statistics | A National Statistics publication for Scotland [Internet]. 2019 [cited 2020 Nov 29]. Available from: https://www.isdscotland.org/Health-Topics/Eye-Care/Publications/2019-10-29/2019-10-29-Ophthalmic-Report.pdf?
15.	Scottish Government. Community eyecare services: review [Internet]. 2017 [cited 2020 Nov 29]. Available from: https://www.gov.scot/publications/community-eyecare-services-review/
16.	Welsh Government. Sensory health (eye care and hearing statistics): April 2017 to March 2019 [Internet]. 2019 [cited 2020 Nov 29]. Available from: https://gov.wales/sensory-health-eye-care-and-hearing-statistics-april-2017-march-2019
17.	Health Service Executive Ireland. Primary Care Eye Services Review Group Report [Internet]. Ireland; 2017 [cited 2020 Oct 10]. Available from: https://www.hse.ie/eng/services/publications/primary/eye-services-review-group-report.pdf
18.	Hong T, Mitchell P, Burlutsky G, Fong CS-U, Rochtchina E, Wang JJ. Visual impairment and subsequent use of support services among older people: longitudinal findings from the Blue Mountains Eye Study. Am J Ophthalmol. 2013;156:393–9.
19.	Napper G, Fricke T, Anjou MD, Jackson AJ. Breaking down barriers to eye care for Indigenous people: a new scheme for delivery of eye care in Victoria. Clin Exp Optom. 2015 Sep 1;98(5):430–4.
20.	Fricke TR, Brand C, Lovett L, Turner NW, Anjou MD, Bentley SA. Lessons learned from a subsidised spectacles scheme aiming to improve eye health in Aboriginal people in Victoria, Australia. Aust Heal Rev. 2021;45(2):194.
21.	Minne-Merri Consultants for the Australian College of Optometry and the Victorian Government Department of Health and Human Services. Evaluation of the Victorian Aboriginal Spectacles Subsidy Scheme [Internet]. 2016 [cited 2020 Oct 14]. Available from: https://www.aco.org.au/wp-content/uploads/2020/04/VASSS-evaluation-report-2017_final.pdf
22.	Maa AY, Wojciechowski B, Hunt K, Dismuke C, Janjua R, Lynch MG. Remote eye care screening for rural veterans with Technology-based Eye Care Services: a quality improvement project. Rural Remote Health. 2017;17:4045.
23.	Borooah S, Grant B, Blaikie A, Styles C, Sutherland S, Forrest G, et al. Using electronic referral with digital imaging between primary and secondary ophthalmic services: A long term prospective analysis of regional service redesign. Eye. 2013 Dec 21;27(3):392–7.
24.	Guymer C, Casson R, Howell C. The AgED Study. Age-related eye disease (AgED) in South Australian general practice: Are we blind to early detection and intervention? Aust J Prim Health. 2017;23(1):75–9.
25.	Cheffins T, Spillman M, Heal C, Kimber D, Brittain M. Evaluating the use of enhanced primary care health assessments by general practices in north Queensland. Aust J Prim Health. 2010;16(3):221–3.
26.	Food and Health Bureau | Department of Health | Government of Hong Kong Special Adminsitrative Region. Report on the review of the elderly health care voucher scheme [Internet]. 2019 [cited 2020 Oct 10]. Available from: https://www.hcv.gov.hk/files/pdf/Review_Report_English.pdf
27.	Turner AW, Mulholland WJ, Taylor HR. Coordination of outreach eye services in remote Australia. Clin Experiment Ophthalmol. 2011;39(4):344–9.
28.	Keel S, Lee PY, Foreman J, van Wijngaarden P, Taylor HR, Dirani M. Participant referral rate in the National Eye Health Survey (NEHS). PLoS One. 2017;12(4):e0174867.
29.	Turner AW, Xie J, Arnold A-L, Dunn RA, Taylor HR. Eye health service access and utilization in the National Indigenous Eye

Health Survey. Clin Experiment Ophthalmol. 2011;39(7):598–603.
30. Dickey H, Ikenwilo D, Norwood P, Watson V, Zangelidis A. Utilisation of eye-care services: The effect of Scotland's free eye examination policy. Health Policy (New York). 2012 Dec;108(2–3):286–93.

Diabetic retinopathy screening or eye examinations

31. Shiu C, Chan P, Lai L, Luk M, Chao D. Reasons for patient defaults from diabetic retinopathy screening in general outpatient clinics (GOPCs) in Hong Kong - A cross sectional study. Hong Kong Pract. 2019;41:3–10.
32. McHugh S, Buckley C, Murphy K, Doherty S, O'Keeffe G, Alade J, et al. Quality-assured screening for diabetic retinopathy delivered in primary care in Ireland: an observational study. Br J Gen Pract. 2013;63(607):e134-40.
33. Rivera-Hernandez M, Leyva B, Keohane LM, Trivedi AN. Quality of Care for White and Hispanic Medicare Advantage Enrollees in the United States and Puerto Rico. JAMA Intern Med. 2016;176(6):787–94.
34. Sia JT, Gan ATL, Soh BP, Fenwick E, Quah J, Sahil T, et al. Rates and predictors of nonadherence to postophthalmic screening tertiary referrals in patients with type 2 diabetes. Transl Vis Sci Technol. 2020;9(6):1–9.
35. An J, Niu F, Turpcu A, Rajput Y, Cheetham TC. Adherence to the American Diabetes Association retinal screening guidelines for population with diabetes in the United States. Ophthalmic Epidemiol. 2018;25(3):257–65.
36. Hwang J, Rudnisky C, Bowen S, Johnson JA. Socioeconomic factors associated with visual impairment and ophthalmic care utilization in patients with type II diabetes. Can J Ophthalmol. 2015;50(2):119–26.
37. Chou C-F, Zhang X, Crews JE, Barker LE, Lee PP, Saaddine JB. Impact of Geographic Density of Eye Care Professionals on Eye Care among Adults with Diabetes. Ophthalmic Epidemiol. 2012 Dec 22;19(6):340–9.
38. Foreman J, Keel S, Xie J, Van Wijngaarden P, Taylor HR, Dirani M. Adherence to diabetic eye examination guidelines in Australia: the National Eye Health Survey. Med J Aust. 2017;206(9):402–6.
39. Tracey M, Racine E, Riordan F, McHugh SM, Kearney PM. Understanding the uptake of a national retinopathy screening programme: An audit of people with diabetes in two large primary care centres. HRB Open Res. 2019 Nov 27;2:17.
40. Gulliford MC, Dodhia H, Chamley M, McCormick K, Mohamed M, Naithani S, et al. Socio-economic and ethnic inequalities in diabetes retinal screening. Diabet Med. 2010;27(3):282–8.
41. Orton E, Forbes-Haley A, Tunbridge L, Cohen S. Equity of uptake of a diabetic retinopathy screening programme in a geographically and socio-economically diverse population. Public Health. 2013;127(9):814–21.
42. Waqar S, Bullen G, Chant S, Salman R, Vaidya B, Ling R. Cost implications, deprivation and geodemographic segmentation analysis of non-attenders (DNA) in an established diabetic retinopathy screening programme. Diabetes Metab Syndr. 2012;6(4):199–202.
43. Virk R, Binns AM, Chambers R, Anderson J. How is the risk of being diagnosed with referable diabetic retinopathy affected by failure to attend diabetes eye screening appointments? Eye. 2021;35(2):477–83.
44. Gibson AA, Humphries J, Gillies M, Nassar N, Colagiuri S. Adherence to eye examination guidelines among individuals with diabetes: An analysis of linked health data. Clin Exp Ohthalmology. 2020;1–10.
45. Eppley SE, Mansberger SL, Ramanathan S, Lowry EA. Characteristics Associated with Adherence to Annual Dilated Eye Examinations among US Patients with Diagnosed Diabetes. Ophthalmology. 2019 Nov;126(11):1492–9.
46. Lundeen EA, Wittenborn J, Benoit SR, Saaddine J. Disparities in Receipt of Eye Exams Among Medicare Part B Fee-for-Service Beneficiaries with Diabetes — United States, 2017. MMWR Morb Mortal Wkly Rep. 2019 Nov 15;68(45):1020–3.
47. Huang OS, Tay WT, Ong PG, Sabanayagam C, Cheng C-Y, Tan GS, et al. Prevalence and determinants of undiagnosed diabetic retinopathy and vision-threatening retinopathy in a multiethnic Asian cohort: the Singapore Epidemiology of Eye Diseases (SEED) study. Br J Ophthalmol. 2015 Dec;99(12):1614–21.
48. Kee LLC, Chong CK, Hwee-lin W, Yean TT. Primary Care Network (PCN) As A Model Of Care For GP Chronic Disease Management. Singapore Fam Physician. 2015;41(2):61–4.
49. Lian JX, McGhee SM, Gangwani RA, Hedley AJ, Lam CLK, Yap MKH, et al. Screening for diabetic retinopathy with or without a copayment in a randomized controlled trial: influence of the inverse care law. Ophthalmology. 2013;120(6):1247–53.
50. Public Health England | Government of the United Kingdom. Screening KPI data summary factsheets | June 2020 - Issue 11 [Internet]. 2020 [cited 2021 Jan 18]. Available from: https://assets.publishing.service.gov.uk/government/uploads/system/uploads/attachment_data/file/897785/Screening_KPI_Summary_Factsheets_June2020_Issue11.pdf
51. Forster AS, Forbes A, Dodhia H, Connor C, Du Chemin A, Sivaprasad S, et al. Changes in Detection of Retinopathy in Type 2 Diabetes in the First 4 Years of a Population-Based Diabetic Eye Screening Program. Diabetes Care. 2013 Sep 1;36(9):2663–9.
52. Scottish Diabetic Retinopathy Screening Collaborative. Diabetic Retinopathy Screening Service Annual Report 2018/19 [Internet]. [cited 2020 Oct 22]. Available from: https://www.ndrs.scot.nhs.uk/wp-content/uploads/2019/11/DRS-Annual-Report-2018.pdf
53. HSC Public Health Agency of Northern Ireland. Diabetic Eye Screening Programme Annual Report 2016-2017 [Internet]. 2017 [cited 2020 Oct 22]. Available from: https://www.publichealth.hscni.net/publications/diabetic-eye-screening-programme-annual-report-2016-2017
54. Public Health Wales. Diabetic Eye Screening Wales Annual Statistical Report 2018-19 [Internet]. 2020 [cited 2020 Oct 22]. Available from: https://phw.nhs.wales/services-and-teams/screening/diabetic-eye-screening-wales-desw/diabetic-eye-screening-wales-annual-statistical-report-2018-191/
55. Yeo ST, Edwards RT, Luzio SD, Charles JM, Thomas RL, Peters JM, et al. Diabetic retinopathy screening: perspectives of people with diabetes, screening intervals and costs of attending screening. Diabet Med. 2012 Jul;29(7):878–85.
56. Looker HC, Nyangoma SO, Cromie DT, Olson JA, Leese GP, Black MW, et al. Rates of referable eye disease in the Scottish National Diabetic Retinopathy Screening Programme. Br J Ophthalmol. 2014 Jun;98(6):790–5.
57. Looker HC, Nyangoma SO, Cromie D, Olson JA, Leese GP, Black M, et al. Diabetic retinopathy at diagnosis of type 2 diabetes in Scotland. Diabetologia. 2012;55:2335–42.
58. National Screening Service Ireland. Diabetic RetinaScreen Programme Report 2013-2015 [Internet]. Ireland; 2017 [cited 2020 Oct 10]. Available from: https://www.diabeticretinascreen.ie/_fileupload/Documents/Diabetic RetinaScreen Programme Report 2013-15 (FINAL web 2) (4).pdf
59. National Screening Service Ireland. Diabetic RetinaScreen Statistical Bulletin 2016-2017 [Internet]. Ireland; 2019 [cited 2020 Oct 10]. Available from: https://www.diabeticretinascreen.ie/_fileupload/Documents/DRS-Statistical-Bulletin-2016-2017-FINAL-

29_11_19.pdf
60. National Screening Service Ireland. Diabetic RetinaScreen Statistical Bulletin 2018-2019. 2019; Available from: https://www.diabeticretinascreen.ie/_fileupload/Documents/DRS-Statistical-Bulletin-2016-2017-FINAL-29_11_19.pdf
61. Joynt KE, Chan DC, Zheng J, Orav EJ, Jha AK. The Impact of Massachusetts Health Care Reform on Access, Quality, and Costs of Care for the Already-Insured. Health Serv Res. 2015 Apr;50(2):599–613.
62. Mamillapalli CK, Prentice JR, Garg AK, Hampsey SL, Bhandari R. Implementation and challenges unique to teleretinal diabetic retinal screening (TDRS) in a private practice setting in the United States. J Clin Transl Endocrinol. 2020 Mar;19:100214.
63. Daskivich LP, Vasquez C, Martinez C, Tseng C-H, Mangione CM. Implementation and Evaluation of a Large-Scale Teleretinal Diabetic Retinopathy Screening Program in the Los Angeles County Department of Health Services. JAMA Intern Med. 2017 May 1;177(5):642–9.
64. Tsan GL, Hoban KL, Jun W, Riedel KJ, Pedersen AL, Hayes J. Assessment of diabetic teleretinal imaging program at the Portland Department of Veterans Affairs Medical Center. J Rehabil Res Dev. 2015;52(2):193–200.
65. Gibson DM. Estimates of the Percentage of US Adults With Diabetes Who Could Be Screened for Diabetic Retinopathy in Primary Care Settings. JAMA Ophthalmol. 2019 Apr 1;137(4):440–4.
66. Gibson DM. Frequency and predictors of missed visits to primary care and eye care providers for annually recommended diabetes preventive care services over a two-year period among U.S. adults with diabetes. Prev Med (Baltim). 2017 Dec;105:257–64.
67. Silva FQ, Adhi M, Wai KM, Olansky L, Lansang MC, Singh RP. Evaluation and Referral of Diabetic Eye Disease in the Endocrinology and Primary Care Office Settings. Ophthalmic Surgery, Lasers Imaging Retin. 2016 Oct;47(10):930–4.
68. Cheung A, Stukel TA, Alter DA, Glazier RH, Ling V, Wang X, et al. Primary Care Physician Volume and Quality of Diabetes Care. Ann Intern Med. 2017 Feb 21;166(4):240–7.
69. Crossland L, Askew D, Ware R, Cranstoun P, Mitchell P, Bryett A, et al. Diabetic Retinopathy Screening and Monitoring of Early Stage Disease in Australian General Practice: Tackling Preventable Blindness within a Chronic Care Model. J Diabetes Res. 2016;2016:1–7.
70. Glasson NM, Crossland LJ, Larkins SL. An Innovative Australian Outreach Model of Diabetic Retinopathy Screening in Remote Communities. J Diabetes Res. 2016;2016:1–10.
71. Ford B, Keay L, Angell B, Hyams S, Mitchell P, Liew G, et al. Quality and targeting of new referrals for ocular complications of diabetes from primary care to a public hospital ophthalmology service in Western Sydney, Australia. Aust J Prim Health. 2020;26(4):293–9.

Glaucoma screening

72. Chua J, Baskaran M, Ong PG, Zheng Y, Wong TY, Aung T, et al. Prevalence, Risk Factors, and Visual Features of Undiagnosed Glaucoma. JAMA Ophthalmol. 2015 Aug 1;133(8):938–46.
73. Adeghate JO, Hark LA, Brown H, Henderer JD, Waisbourd M, Molineaux J, et al. Philadelphia glaucoma detection and treatment project: ocular outcomes and adherence to follow-up at a single health centre. Can J Ophthalmol. 2019;54:717–22.
74. Hark LA, Leiby BE, Waisbourd M, Myers JS, Fudemberg SJ, Mantravadi A V, et al. Adherence to Follow-up Recommendations Among Individuals in the Philadelphia Glaucoma Detection and Treatment Project. J Glaucoma. 2017;26:697–701.
75. Zhao D, Guallar E, Gajwani P, Swenor B, Crews J, Saaddine J, et al. Optimizing Glaucoma Screening in High-Risk Population: Design and 1-Year Findings of the Screening to Prevent (SToP) Glaucoma Study. Am J Ophthalmol. 2017 Aug;180:18–28.
76. Zhao D, Guallar E, Bowie J V, Swenor B, Gajwani P, Kanwar N, et al. Improving Follow-up and Reducing Barriers for Eye Screenings in Communities: The SToP Glaucoma Study. Am J Ophthalmol. 2018 Apr;188:19–28.
77. Sii S, Nasser A, Loo CY, Croghan C, Rotchford A, Agarwal PK. The impact of SIGN glaucoma guidelines on false-positive referrals from community optometrists in Central Scotland. Br J Ophthalmol. 2019 Mar;103(3):369–73.
78. El-Assal K, Foulds J, Dobson S, Sanders R. A comparative study of glaucoma referrals in Southeast Scotland: effect of the new general ophthalmic service contract, Eyecare integration pilot programme and NICE guidelines. BMC Ophthalmol. 2015 Dec 7;15(1):172.
79. Ratnarajan G, Newsom W, Vernon SA, Fenerty C, Henson D, Spencer F, et al. The effectiveness of schemes that refine referrals between primary and secondary care—the UK experience with glaucoma referrals: the Health Innovation & Education Cluster (HIEC) Glaucoma Pathways Project. BMJ Open. 2013 Jul;3(7):e002715.
80. Ratnarajan G, Kean J, French K, Parker M, Bourne R. The false negative rate and the role for virtual review in a nationally evaluated glaucoma referral refinement scheme. Ophthalmic Physiol Opt. 2015 Sep;35(5):577–81.
81. Bourne RRA, French KA, Chang L, Borman AD, Hingorani M, Newsom WD. Can a community optometrist-based referral refinement scheme reduce false-positive glaucoma hospital referrals without compromising quality of care? The community and hospital allied network glaucoma evaluation scheme (CHANGES). Eye. 2010 May 31;24(5):881–7.
82. Chawla A, Patel I, Yuen C, Fenerty C. Patterns of adherence to NICE Glaucoma Guidance in two different service delivery models. Eye. 2012 Nov;26(11):1412–7.
83. Trikha S, Macgregor C, Jeffery M, Kirwan J. The Portsmouth-based glaucoma refinement scheme: a role for virtual clinics in the future? Eye. 2012 Oct 6;26(10):1288–94.
84. Roberts HW, Rughani K, Syam P, Dhingra S, Ramirez-Florez S. The Peterborough Scheme for Community Specialist Optometrists in Glaucoma: Results of 4 Years of a Two-Tiered Community-Based Assessment and Follow-up Service. Curr Eye Res. 2015 Jul 3;40(7):690–6.
85. Parkins DJ, Edgar DF. Comparison of the effectiveness of two enhanced glaucoma referral schemes. Ophthalmic Physiol Opt. 2011 Jul 1;31(4):343–52.
86. Devarajan N, Williams GS, Hopes M, O'Sullivan D, Jones D. The Carmarthenshire Glaucoma Referral Refinement Scheme, a safe and efficient screening service. Eye. 2011 Jan 22;25(1):43–9.
87. Vernon SA, Adair A. Shared care in glaucoma: a national study of secondary care lead schemes in England. Eye. 2010 Feb 5;24(2):265–9.
88. Fung SSM, Lemer C, Russell RA, Malik R, Crabb DP. Are practical recommendations practiced? A national multi-centre cross-sectional study on frequency of visual field testing in glaucoma. Br J Ophthalmol. 2013;97:843–7.
89. Zangerl B, Hayen A, Mitchell P, Jamous KF, Stapleton F, Kalloniatis M. Therapeutic endorsement enhances compliance with national glaucoma guidelines in Australian and New Zealand optometrists. Ophthalmic Physiol Opt. 2015 Mar;35(2):212–24.

Cataract

90. Penrose L, Roe Y, Johnson NA. Process redesign of a surgical pathway improves access to cataract surgery for Aboriginal and Torres Strait Islander people in South East Queensland. Aust J Prim Health. 2018;24:135–40.
91. Chua J, Lim B, Fenwick EK, Gan ATL, Tan AG, Lamoureux E, et al. Prevalence, Risk Factors, and Impact of Undiagnosed Visually Significant Cataract: The Singapore Epidemiology of Eye Diseases Study. Pan C-W, editor. PLoS One. 2017 Jan 27;12(1):e0170804.
92. Bowes OMB, Shah P, Rana M, Farrell S, Rajan MS. Quality indicators in a community optometrist led cataract shared care scheme. Ophthalmic Physiol Opt. 2018;38:183–92.
93. Voyatzis G, Roberts HW, Keenan J, Rajan MS. Cambridgeshire cataract shared care model: community optometrist-delivered postoperative discharge scheme. Br J Ophthalmol. 2014;98:760–4.
94. Coronini-Cronberg S, Lee H, Darzi A, Smith P. Evaluation of clinical threshold policies for cataract surgery among English commissioners. J Health Serv Res Policy. 2012;17:241–7.
95. Lin T, Xu M, Hooper PL. Adequacy of the ophthalmology workforce under Ontario's Local Health Integration Networks. Can J Ophthalmol. 2016;51:142–6.
96. Pershing S, Morrison DE, Hernandez-Boussard T. Cataract Surgery Complications and Revisit Rates Among Three States. Am J Ophthalmol. 2016;171:130–8.

Low vision

97. Scottish Government. Low vision service provision in Scotland: review [Internet]. 2017 [cited 2020 Nov 29]. Available from: https://www.gov.scot/publications/review-low-vision-service-provision-scotland/pages/6/
98. Ryan B, Khadka J, Bunce C, Court H. Effectiveness of the community-based Low Vision Service Wales: a long-term outcome study. Br J Ophthalmol. 2013;97:487–91.
99. Court H, Ryan B, Bunce C, Margrain TH. How effective is the new community-based Welsh low vision service?. Br J Ophthalmol. 2011;95:178–84.
100. Ryan B, White S, Wild J, Court H, Margrain TH. The newly established primary care based Welsh Low Vision Service is effective and has improved access to low vision services in Wales. Ophthalmic Physiol Opt. 2010;30:358–64.
101. Duckworth S. Stocktake and Needs Analysis of Low Vision Services in New Zealand Prepared for Ministry of Health Manatū Hauora [Internet]. Ministry of Health, New Zealand Government; 2015 [cited 2020 Oct 9]. Available from: https://www.health.govt.nz/publication/stocktake-and-needs-analysis-low-vision-services-new-zealand

Age-related macular degeneration

102. Li B, Powell A-M, Hooper PL, Sheidow TG. Prospective Evaluation of Teleophthalmology in Screening and Recurrence Monitoring of Neovascular Age-Related Macular Degeneration. JAMA Ophthalmol. 2015 Mar 1;133(3):276–82.

List of references cited in Supplementary Material

1. Clarke EL, Evans JR, Smeeth L. Community screening for visual impairment in older people. Cochrane Database Syst Rev. 2018 Feb 20;2018(2).
2. Wilson BJ, Courage S, Bacchus M, Dickinson JA, Klarenbach S, Jaramillo Garcia A, et al. Screening for impaired vision in community-dwelling adults aged 65 years and older in primary care settings. Can Med Assoc J. 2018;190(19):E588–94.
3. Chou R, Dana T, Bougatsos C, Grusing S, Blazina I. Screening for impaired visual acuity in older adults: Updated evidence report and systematic review for the US preventive services task force. J Am Med Assoc. 2016;315(9):915–33.
4. Siu AL, Bibbins-Domingo K, Grossman DC, Baumann LC, Davidson KW, Ebell M, et al. Screening for impaired visual acuity in older adults: US preventive services task force recommendation statement. J Am Med Assoc. 2016 Mar 1;315(9):908–14.
5. Friedman DS, Cassard SD, Williams SK, Baldonado K, O'Brien RW, Gower EW. Outcomes of a vision screening program for underserved populations in the united states. Ophthalmic Epidemiol. 2013;20(4):201–11.
6. Shiu C, Chan P, Lai L, Luk M, Chao D. Reasons for patient defaults from diabetic retinopathy screening in general outpatient clinics (GOPCs) in Hong Kong - A cross sectional study. Hong Kong Pract. 2019;41:3–10.
7. McHugh S, Buckley C, Murphy K, Doherty S, O'Keeffe G, Alade J, et al. Quality-assured screening for diabetic retinopathy delivered in primary care in Ireland: an observational study. Br J Gen Pract. 2013;63(607):e134-40.
8. Rivera-Hernandez M, Leyva B, Keohane LM, Trivedi AN. Quality of Care for White and Hispanic Medicare Advantage Enrollees in the United States and Puerto Rico. JAMA Intern Med. 2016;176(6):787–94.
9. Sia JT, Gan ATL, Soh BP, Fenwick E, Quah J, Sahil T, et al. Rates and predictors of nonadherence to postophthalmic screening tertiary referrals in patients with type 2 diabetes. Transl Vis Sci Technol. 2020;9(6):1–9.
10. An J, Niu F, Turpcu A, Rajput Y, Cheetham TC. Adherence to the American Diabetes Association retinal screening guidelines for population with diabetes in the United States. Ophthalmic Epidemiol. 2018;25(3):257–65.
11. Hwang J, Rudnisky C, Bowen S, Johnson JA. Socioeconomic factors associated with visual impairment and ophthalmic care utilization in patients with type II diabetes. Can J Ophthalmol. 2015;50(2):119–26.
12. Chou CF, Zhang X, Crews JE, Barker LE, Lee PP, Saaddine JB. Impact of Geographic Density of Eye Care Professionals on Eye Care among Adults with Diabetes. Ophthalmic Epidemiol. 2012 Dec 22;19(6):340–9.
13. Foreman J, Keel S, Xie J, Van Wijngaarden P, Taylor HR, Dirani M. Adherence to diabetic eye examination guidelines in Australia: the National Eye Health Survey. Med J Aust. 2017;206(9):402–6.
14. Tracey M, Racine E, Riordan F, McHugh SM, Kearney PM. Understanding the uptake of a national retinopathy screening programme: An audit of people with diabetes in two large primary care centres. HRB Open Res. 2019 Nov 27;2:17.
15. Gulliford MC, Dodhia H, Chamley M, McCormick K, Mohamed M, Naithani S, et al. Socio-economic and ethnic inequalities in diabetes retinal screening. Diabet Med. 2010;27(3):282–8.
16. Orton E, Forbes-Haley A, Tunbridge L, Cohen S. Equity of uptake of a diabetic retinopathy screening programme in a geographically and socio-economically diverse population. Public Health. 2013;127(9):814–21.
17. Waqar S, Bullen G, Chant S, Salman R, Vaidya B, Ling R. Cost implications, deprivation and geodemographic segmentation analysis of non-attenders (DNA) in an established diabetic retinopathy screening programme. Diabetes Metab Syndr. 2012;6(4):199–202.
18. Virk R, Binns AM, Chambers R, Anderson J. How is the risk of being diagnosed with referable diabetic retinopathy affected by failure to attend diabetes eye screening appointments? Eye. 2021;35(2):477–83.
19. Gibson AA, Humphries J, Gillies M, Nassar N, Colagiuri S. Adherence to eye examination guidelines among individuals with diabetes: An analysis of linked health data. Clin Exp Ohthalmology. 2020;1–10.
20. Eppley SE, Mansberger SL, Ramanathan S, Lowry EA. Characteristics Associated with Adherence to Annual Dilated Eye Examinations among US Patients with Diagnosed Diabetes. Ophthalmology. 2019 Nov;126(11):1492–9.
21. Lundeen EA, Wittenborn J, Benoit SR, Saaddine J. Disparities in Receipt of Eye Exams Among Medicare Part B Fee-for-Service Beneficiaries with Diabetes — United States, 2017. MMWR Morb Mortal Wkly Rep. 2019 Nov 15;68(45):1020–3.
22. Balegamire S, Aubin MJ, Curcio CL, Alvarado B, Guerra RO, Ylli A, et al. Factors Associated With Visual Impairment and Eye Care Utilization: The International Mobility in Aging Study. J Aging Health. 2018;30(9):1369–88.
23. Iliffe S, Kharicha K, Harari D, Swift C, Gillmann G, Stuck AE. Health risk appraisal in older people 6: factors associated with self-reported poor vision and uptake of eye tests in older people. BMC Fam Pract. 2013;14:130.
24. Morales LS, Varma R, Paz SH, Lai MY, Mazhar K, Andersen RM, et al. Self-reported use of eye care among Latinos: the Los Angeles Latino Eye Study. Ophthalmology. 2010;117(2):207–15.
25. Gupta P, Majithia S, Fenwick EK, Gan ATL, Tham YC, Poh S, et al. Rates and Determinants of Eyecare Utilization and Eyeglass Affordability Among Individuals With Visual Impairment in a Multi-Ethnic Population-Based Study in Singapore. Transl Vis Sci Technol [Internet]. 2020 Apr 18;9(5):11. Available from: https://tvst.arvojournals.org/article.aspx?articleid=2765019
26. Centers for Disease Control and Prevention (CDC). Eye-care utilization among women aged > or =40 years with eye diseases--19 states, 2006-2008. Vol. 59, Morbidity and mortality weekly report. 2010.
27. Zhao D, Guallar E, Gajwani P, Swenor B, Crews J, Saaddine J, et al. Optimizing Glaucoma Screening in High-Risk Population: Design and 1-Year Findings of the Screening to Prevent (SToP) Glaucoma Study. Am J Ophthalmol. 2017 Aug;180:18–28.
28. Zhao D, Guallar E, Bowie J V, Swenor B, Gajwani P, Kanwar N, et al. Improving Follow-up and Reducing Barriers for Eye Screenings in Communities: The SToP Glaucoma Study. Am J Ophthalmol. 2018;188:19–28.
29. Adeghate JO, Hark LA, Brown H, Henderer JD, Waisbourd M, Molineaux J, et al. Philadelphia glaucoma detection and treatment project: ocular outcomes and adherence to follow-up at a single health centre. Can J Ophthalmol. 2019;54:717–22.
30. Hark LA, Leiby BE, Waisbourd M, Myers JS, Fudemberg SJ, Mantravadi A V, et al. Adherence to Follow-up Recommendations Among Individuals in the Philadelphia Glaucoma Detection and Treatment Project. J Glaucoma. 2017;26:697–701.
31. Huang OS, Tay WT, Ong PG, Sabanayagam C, Cheng CY, Tan GS, et al. Prevalence and determinants of undiagnosed diabetic retinopathy and vision-threatening retinopathy in a multiethnic Asian cohort: the Singapore Epidemiology of Eye Diseases (SEED) study. Br J Ophthalmol. 2015 Dec;99(12):1614–21.
32. Chua J, Baskaran M, Ong PG, Zheng Y, Wong TY, Aung T, et al. Prevalence, Risk Factors, and Visual Features of Undiagnosed Glaucoma. JAMA Ophthalmol. 2015 Aug 1;133(8):938–46.
33. Chua J, Lim B, Fenwick EK, Gan ATL, Tan AG, Lamoureux E, et al. Prevalence, Risk Factors, and Impact of Undiagnosed Visually

Significant Cataract: The Singapore Epidemiology of Eye Diseases Study. Pan CW, editor. PLoS One. 2017 Jan 27;12(1):e0170804.
34. An J, Niu F, Turpcu A, Rajput Y, Cheetham TC, An J. Adherence to the American Diabetes Association retinal screening guidelines for population with diabetes in the United States Adherence to the American Diabetes Association retinal screening guidelines for population with diabetes in the United States. Ophthalmic Epidemiol [Internet]. 2018;25(3):257–65. Available from: https://doi.org/10.1080/09286586.2018.1424344
35. Eppley SE, Mansberger SL, Ramanathan S, Lowry EA. Characteristics Associated with Adherence to Annual Dilated Eye Examinations among US Patients with Diagnosed Diabetes. Ophthalmology [Internet]. 2019 Nov;126(11):1492–9. Available from: NS -
36. Looker HC, Nyangoma SO, Cromie D, Olson JA, Leese GP, Black M, et al. Diabetic retinopathy at diagnosis of type 2 diabetes in Scotland. Diabetologia. 2012;55:2335–42.
37. Northern Ireland Statistics and Research Organisation. General Ophthalmic Services Statistics for Northern Ireland, Annual Statistics 2019/20 [Internet]. Belfast: Business Services Organisation Family Practitioner Services Information Unit; 2020 [cited 2020 Nov 29]. Available from: https://hscbusiness.hscni.net/services/3175.htm
38. Public Health Scotland. General Ophthalmic Services [Internet]. Edinburgh: Public Health Scotland; 2020 [cited 2020 Oct 21]. Available from: https://www.isdscotland.org/Health-Topics/Eye-Care/General-Ophthalmic-Services/
39. Population Health Directorate | Health and Social Care | Scottish Government. Community eyecare services: review [Internet]. Edinburgh: Scottish Government; 2017 [cited 2020 Nov 29]. Available from: https://www.gov.scot/publications/community-eyecare-services-review/
40. Statistics for Wales. Sensory health (eye care and hearing statistics): April 2017 to March 2019 [Internet]. Welsh Government; 2019 [cited 2020 Nov 29]. Available from: https://gov.wales/sensory-health-eye-care-and-hearing-statistics-april-2017-march-2019
41. Lawrenson J, Lorencatto F, Burr J, Bunce C, Jj F, Aluko P, et al. Interventions to increase attendance for diabetic retinopathy screening. 2018;(1).
42. NHS Digital. General Ophthalmic Services Activity Statistics England, year ending 31 March 2020 - NHS Digital [Internet]. 2020 [cited 2020 Nov 29]. Available from: https://digital.nhs.uk/data-and-information/publications/statistical/general-ophthalmic-services-activity-statistics/england-year-ending-31-march-2020
43. Information Services Division. General Ophthalmic Services Statistics | A National Statistics publication for Scotland [Internet]. Public Health Scotland; 2019 [cited 2020 Nov 29]. Available from: https://www.isdscotland.org/Health-Topics/Eye-Care/Publications/2019-10-29/2019-10-29-Ophthalmic-Report.pdf?
44. Gibson DM. Estimates of the Percentage of US Adults With Diabetes Who Could Be Screened for Diabetic Retinopathy in Primary Care Settings. JAMA Ophthalmol. 2019 Apr 1;137(4):440–4.
45. Foreman J, Xie J, Keel S, van Wijngaarden P, Sandhu SS, Ang GS, et al. The Prevalence and Causes of Vision Loss in Indigenous and Non-Indigenous Australians: The National Eye Health Survey. Ophthalmology [Internet]. 2017;124(12 PG):1743–52. Available from: NS -
46. Waqar S, Bullen G, Chant S, Salman R, Vaidya B, Ling R. Cost implications, deprivation and geodemographic segmentation analysis of non-attenders (DNA) in an established diabetic retinopathy screening programme. Diabetes Metab Syndr Clin Res Rev. 2012;6(4):199–202.
47. Dickey H, Ikenwilo D, Norwood P, Watson V, Zangelidis A. Utilisation of eye-care services: The effect of Scotland's free eye examination policy. Health Policy (New York). 2012 Dec;108(2–3):286–93.
48. Duckworth S. Stocktake and Needs Analysis of Low Vision Services in New Zealand Prepared for Ministry of Health Manatū Hauora [Internet]. Ministry of Health, New Zealand Government; 2015 [cited 2020 Oct 9]. Available from: https://www.health.govt.nz/publication/stocktake-and-needs-analysis-low-vision-services-new-zealand
49. Scottish Government. Low vision service provision in Scotland: review [Internet]. 2017 [cited 2020 Nov 29]. Available from: https://www.gov.scot/publications/review-low-vision-service-provision-scotland/pages/6/
50. Coronini-Cronberg S, Lee H, Darzi A, Smith P. Evaluation of clinical threshold policies for cataract surgery among English commissioners. J Health Serv Res Policy. 2012;17:241–7.
51. Public Health Agency. Diabetic Eye Screening Programme Annual Report 2016-2017 [Internet]. Belfast: Public Health Agency; 2017 [cited 2020 Oct 22]. Available from: https://www.publichealth.hscni.net/publications/diabetic-eye-screening-programme-annual-report-2016-2017
52. Public Health Wales. Diabetic Eye Screening Wales Annual Statistical Report 2018-19 [Internet]. Rhondda Cynon Taff: Public Health Wales; 2020 [cited 2020 Oct 22]. Available from: https://phw.nhs.wales/services-and-teams/screening/diabetic-eye-screening-wales-desw/diabetic-eye-screening-wales-annual-statistical-report-2018-191/
53. Turner AW, Xie J, Arnold AL, Dunn RA, Taylor HR. Eye health service access and utilization in the National Indigenous Eye Health Survey. Clin Experiment Ophthalmol. 2011;39(7):598–603.
54. Glasson NM, Crossland LJ, Larkins SL. An Innovative Australian Outreach Model of Diabetic Retinopathy Screening in Remote Communities. J Diabetes Res. 2016;2016:1–10.
55. Penrose L, Roe Y, Johnson NA. Process redesign of a surgical pathway improves access to cataract surgery for Aboriginal and Torres Strait Islander people in South East Queensland. Aust J Prim Health. 2018;24:135–40.
56. Fricke TR, Brand C, Lovett L, Turner NW, Anjou MD, Bentley SA. Lessons learned from a subsidised spectacles scheme aiming to improve eye health in Aboriginal people in Victoria, Australia. Aust Heal Rev. 2021;45(2):194.
57. Napper G, Fricke T, Anjou MD, Jackson AJ. Breaking down barriers to eye care for Indigenous people: a new scheme for delivery of eye care in Victoria. Clin Exp Optom. 2015 Sep 1;98(5):430–4.
58. Fricke T, Consultants MM. Evaluation of the Victorian Aboriginal Spectacles Subsidy Scheme [Internet]. Melbourne: Australian College of Optometry and the Victorian Government Department of Health and Human Services; 2016 [cited 2020 Oct 14]. Available from: https://www.aco.org.au/wp-content/uploads/2020/04/VASSS-evaluation-report-2017_final.pdf
59. Ratnarajan G, Kean J, French K, Parker M, Bourne R. The false negative rate and the role for virtual review in a nationally evaluated glaucoma referral refinement scheme. Ophthalmic Physiol Opt. 2015 Sep;35(5):577–81.
60. Bourne RRA, French KA, Chang L, Borman AD, Hingorani M, Newsom WD. Can a community optometrist-based referral refinement scheme reduce false-positive glaucoma hospital referrals without compromising quality of care? The community and hospital allied network glaucoma evaluation scheme (CHANGES). Eye. 2010 May 31;24(5):881–7.

61. Ratnarajan G, Newsom W, Vernon SA, Fenerty C, Henson D, Spencer F, et al. The effectiveness of schemes that refine referrals between primary and secondary care—the UK experience with glaucoma referrals: the Health Innovation & Education Cluster (HIEC) Glaucoma Pathways Project. BMJ Open. 2013 Jul;3(7):e002715.
62. Chawla A, Patel I, Yuen C, Fenerty C. Patterns of adherence to NICE Glaucoma Guidance in two different service delivery models. Eye. 2012 Nov;26(11):1412–7.
63. Trikha S, Macgregor C, Jeffery M, Kirwan J. The Portsmouth-based glaucoma refinement scheme: a role for virtual clinics in the future? Eye. 2012 Oct 6;26(10):1288–94.
64. Roberts HW, Rughani K, Syam P, Dhingra S, Ramirez-Florez S. The Peterborough Scheme for Community Specialist Optometrists in Glaucoma: Results of 4 Years of a Two-Tiered Community-Based Assessment and Follow-up Service. Curr Eye Res. 2015 Jul 3;40(7):690–6.
65. Parkins DJ, Edgar DF. Comparison of the effectiveness of two enhanced glaucoma referral schemes. Ophthalmic Physiol Opt. 2011 Jul 1;31(4):343–52.
66. Devarajan N, Williams GS, Hopes M, O'Sullivan D, Jones D. The Carmarthenshire Glaucoma Referral Refinement Scheme, a safe and efficient screening service. Eye. 2011 Jan 22;25(1):43–9.
67. Vernon SA, Adair A. Shared care in glaucoma: a national study of secondary care lead schemes in England. Eye. 2010 Feb 5;24(2):265–9.
68. Sii S, Nasser A, Loo CY, Croghan C, Rotchford A, Agarwal PK. The impact of SIGN glaucoma guidelines on false-positive referrals from community optometrists in Central Scotland. Br J Ophthalmol. 2019 Mar;103(3):369–73.
69. El-Assal K, Foulds J, Dobson S, Sanders R. A comparative study of glaucoma referrals in Southeast Scotland: effect of the new general ophthalmic service contract, Eyecare integration pilot programme and NICE guidelines. BMC Ophthalmol. 2015 Dec 7;15(1):172.
70. Voyatzis G, Roberts HW, Keenan J, Rajan MS. Cambridgeshire cataract shared care model: community optometrist-delivered postoperative discharge scheme. Br J Ophthalmol. 2014;98:760–4.
71. Bowes OMB, Shah P, Rana M, Farrell S, Rajan MS. Quality indicators in a community optometrist led cataract shared care scheme. Ophthalmic Physiol Opt. 2018;38:183–92.
72. Health Service Executive Ireland. Primary Care Eye Services Review Group Report [Internet]. Ireland; 2017 [cited 2020 Oct 10]. Available from: https://www.hse.ie/eng/services/publications/primary/eye-services-review-group-report.pdf
73. Food and Health Bureau | Department of Health. Report on the review of the elderly health care voucher scheme [Internet]. Government of Hong Kong Special Administrative Region; 2019 [cited 2020 Oct 10]. Available from: https://www.hcv.gov.hk/files/pdf/Review_Report_English.pdf
74. Gibson DM. Frequency and predictors of missed visits to primary care and eye care providers for annually recommended diabetes preventive care services over a two-year period among U.S. adults with diabetes. Prev Med (Baltim). 2017 Dec;105:257–64.
75. Silva FQ, Adhi M, Wai KM, Olansky L, Lansang MC, Singh RP. Evaluation and Referral of Diabetic Eye Disease in the Endocrinology and Primary Care Office Settings. Ophthalmic Surgery, Lasers Imaging Retin. 2016 Oct;47(10):930–4.
76. Ford B, Keay L, Angell B, Hyams S, Mitchell P, Liew G, et al. Quality and targeting of new referrals for ocular complications of diabetes from primary care to a public hospital ophthalmology service in Western Sydney, Australia. Aust J Prim Health. 2020;26(4):293–9.
77. Guymer C, Casson R, Howell C. The AgED Study. Age-related eye disease (AgED) in South Australian general practice: Are we blind to early detection and intervention? Aust J Prim Health. 2017;23(1):75–9.
78. Cheffins T, Spillman M, Heal C, Kimber D, Brittain M. Evaluating the use of enhanced primary care health assessments by general practices in north Queensland. Aust J Prim Health. 2010;16(3):221–3.
79. Crossland L, Askew D, Ware R, Cranstoun P, Mitchell P, Bryett A, et al. Diabetic Retinopathy Screening and Monitoring of Early Stage Disease in Australian General Practice: Tackling Preventable Blindness within a Chronic Care Model. J Diabetes Res. 2016;2016:1–7.
80. Kee LLC, Chong CK, Hwee-lin W, Yean TT. Primary Care Network (PCN) As A Model Of Care For GP Chronic Disease Management. Singapore Fam Physician. 2015;41(2):61–4.
81. Forster AS, Forbes A, Dodhia H, Connor C, Du Chemin A, Sivaprasad S, et al. Changes in Detection of Retinopathy in Type 2 Diabetes in the First 4 Years of a Population-Based Diabetic Eye Screening Program. Diabetes Care. 2013 Sep 1;36(9):2663–9.
82. Looker HC, Nyangoma SO, Cromie D, Olson JA, Leese GP, Black M, et al. Diabetic retinopathy at diagnosis of type 2 diabetes in Scotland. Diabetologia. 2012;55:2335–42.
83. Looker HC, Nyangoma SO, Cromie DT, Olson JA, Leese GP, Black MW, et al. Rates of referable eye disease in the Scottish National Diabetic Retinopathy Screening Programme. Br J Ophthalmol. 2014 Jun;98(6):790–5.
84. National Screening Service. Diabetic RetinaScreen Programme Report 2013-2015 [Internet]. Ireland: Health Services Executive; 2017 [cited 2020 Oct 10]. Available from: https://www.diabeticretinascreen.ie/_fileupload/Documents/Diabetic RetinaScreen Programme Report 2013-15 (FINAL web 2) (4).pdf
85. National Screening Service. Diabetic RetinaScreen Statistical Bulletin 2016-2017 [Internet]. Ireland: Health Services Executive; 2019 [cited 2020 Oct 10]. Available from: https://www.diabeticretinascreen.ie/_fileupload/Documents/DRS-Statistical-Bulletin-2016-2017-FINAL-29_11_19.pdf
86. National Screening Service Ireland. Diabetic RetinaScreen Statistical Bulletin 2018-2019. 2019; Available from: https://www.diabeticretinascreen.ie/_fileupload/Documents/DRS-Statistical-Bulletin-2016-2017-FINAL-29_11_19.pdf
87. Li B, Powell AM, Hooper PL, Sheidow TG. Prospective Evaluation of Teleophthalmology in Screening and Recurrence Monitoring of Neovascular Age-Related Macular Degeneration. JAMA Ophthalmol. 2015 Mar 1;133(3):276–82.
88. Borooah S, Grant B, Blaikie A, Styles C, Sutherland S, Forrest G, et al. Using electronic referral with digital imaging between primary and secondary ophthalmic services: A long term prospective analysis of regional service redesign. Eye. 2013 Dec 21;27(3):392–7.
89. Zangerl B, Hayen A, Mitchell P, Jamous KF, Stapleton F, Kalloniatis M. Therapeutic endorsement enhances compliance with national glaucoma guidelines in Australian and New Zealand optometrists. Ophthalmic Physiol Opt. 2015 Mar;35(2):212–24.
90. Fung SSM, Lemer C, Russell RA, Malik R, Crabb DP. Are practical recommendations practiced? A national multi-centre cross-sectional study on frequency of visual field testing in glaucoma. Br J Ophthalmol. 2013;97:843–7.
91. Cheung A, Stukel TA, Alter DA, Glazier RH, Ling V, Wang X, et al. Primary Care Physician Volume and Quality of Diabetes Care. Ann Intern Med. 2017 Feb 21;166(4):240–7.

92. Pershing S, Morrison DE, Hernandez-Boussard T. Cataract Surgery Complications and Revisit Rates Among Three States. Am J Ophthalmol. 2016;171:130–8.
93. Ryan B, White S, Wild J, Court H, Margrain TH. The newly established primary care based Welsh Low Vision Service is effective and has improved access to low vision services in Wales. Ophthalmic Physiol Opt. 2010;30:358–64.
94. Ryan B, Khadka J, Bunce C, Court H. Effectiveness of the community-based Low Vision Service Wales: a long-term outcome study. Br J Ophthalmol. 2013;97:487–91.
95. Court H, Ryan B, Bunce C, Margrain TH. How effective is the new community-based Welsh low vision service?. Br J Ophthalmol. 2011;95:178–84.
96. Yeo ST, Edwards RT, Luzio SD, Charles JM, Thomas RL, Peters JM, et al. Diabetic retinopathy screening: perspectives of people with diabetes, screening intervals and costs of attending screening. Diabet Med. 2012 Jul;29(7):878–85.
97. Maa AY, Wojciechowski B, Hunt K, Dismuke C, Janjua R, Lynch MG. Remote eye care screening for rural veterans with Technology-based Eye Care Services: a quality improvement project. Rural Remote Health. 2017;17:4045.
98. Lian JX, McGhee SM, Gangwani RA, Hedley AJ, Lam CLK, Yap MKH, et al. Screening for diabetic retinopathy with or without a copayment in a randomized controlled trial: influence of the inverse care law. Ophthalmology. 2013;120(6):1247–53.
99. Ministry of Health | New Zealand Government. Diabetic Retinal Screening, Grading, Monitoring and Referral Guidance [Internet]. Wellington, New Zealand; 2016 [cited 2020 Oct 9]. Available from: https://www.health.govt.nz/system/files/documents/publications/diabetic-retinal-screening-grading-monitoring-referral-guidance-mar16.pdf
100. Ministry of Health, Government NZ. Equipment for people who are blind or have reduced vision [Internet]. Wellington: New Zealand Government; 2019 [cited 2020 Oct 9]. Available from: https://www.health.govt.nz/your-health/services-and-support/disability-services/types-disability-support/hearing-and-vision-services/equipment-people-who-are-blind-or-have-reduced-vision
101. Ministry of Health | New Zealand Government. Contact lens subsidy | Ministry of Health NZ [Internet]. 2019 [cited 2020 Oct 9]. Available from: https://www.health.govt.nz/new-zealand-health-system/claims-provider-payments-and-entitlements/contact-lens-subsidy
102. Work and Income | Ministry of Social Development | New Zealand Government. Glasses [Internet]. [cited 2020 Oct 9]. Available from: https://www.workandincome.govt.nz/eligibility/health-and-disability/glasses.html#null
103. Department of Health | Australian Government. MBS Online | Medicare Benefits Schedule [Internet]. [cited 2020 Oct 14]. Available from: http://www.mbsonline.gov.au/internet/mbsonline/publishing.nsf/Content/Home
104. Diabetes Australia. KeepSight [Internet]. [cited 2020 Oct 14]. Available from: https://www.keepsight.org.au/about
105. Department of Veterans' Affairs | Australian Government. Optical services [Internet]. 2019 [cited 2020 Oct 14]. Available from: https://www.dva.gov.au/health-and-treatment/injury-or-health-treatments/health-services/optical-services
106. Queensland Government. Spectacles [Internet]. [cited 2020 Oct 14]. Available from: https://www.qld.gov.au/health/support/equipment/types/spectacles
107. Vision Australia. NSW Spectacles Program [Internet]. [cited 2020 Oct 14]. Available from: https://www.visionaustralia.org/referral/benefits-and-funding/nsw-spectacles
108. NSW Government. Blindness and Low Vision Assistive Technology Funding Criteria [Internet]. 2018 [cited 2020 Nov 14]. Available from: https://www.enable.health.nsw.gov.au/__data/assets/pdf_file/0003/262407/blindness-and-low-vision-assistive-technology-funding-criteria.pdf
109. ACT Revenue Office | ACT Government. Spectacles Subsidy Scheme [Internet]. [cited 2020 Oct 14]. Available from: https://www.revenue.act.gov.au/community-assistance/spectacles-subsidy-scheme
110. ACT Revenue Office | ACT Government. ACT Spectacles Subsidy Scheme & Seniors Spectacles Scheme Policy Paper [Internet]. [cited 2020 Oct 14]. Available from: https://www.cmtedd.act.gov.au/__data/assets/pdf_file/0009/1134549/ACT-Spectacles-Subsidy-Scheme-and-Seniors-Spectacles-Scheme-Policy-Paper.pdf
111. ACT Revenue Office | ACT Government. Low Vision Aids Scheme [Internet]. [cited 2020 Oct 14]. Available from: https://www.revenue.act.gov.au/community-assistance/low-vision-aids-scheme
112. Victorian State Government. Victorian Eyecare Service [Internet]. [cited 2020 Oct 14]. Available from: https://www2.health.vic.gov.au/ageing-and-aged-care/supporting-independent-living/victorian-eye-care-service
113. Australian College of Optometry. Victorian Eyecare Service [Internet]. Melbourne: Australian College of Optometry; [cited 2020 Oct 14]. Available from: https://www.aco.org.au/victorian-eyecare-service/
114. Northern Territory Government. Spectacle concessions [Internet]. [cited 2020 Oct 14]. Available from: https://nt.gov.au/community/concessions-and-payments/nt-concession-scheme/concessions/spectacles
115. South Australian Government. GlassesSA [Internet]. [cited 2020 Oct 14]. Available from: https://www.sa.gov.au/topics/care-and-support/concessions-and-grants/concessions/GlassesSA
116. Department of Health | Government of Western Australia. Spectacle Subsidy Scheme [Internet]. [cited 2020 Oct 14]. Available from: https://healthywa.wa.gov.au/Articles/S_T/Spectacle-Subsidy-Scheme
117. Tasmanian Government. Tasmanian Government Concessions Guide [Internet]. [cited 2020 Oct 14]. Available from: http://www.dpac.tas.gov.au/__data/assets/pdf_file/0004/121558/Tasmanian_Government_Concessions_Guide_2011-12.pdf
118. Ministerial Committee on Ageing. Project Silver Screen: I Feel Young SG [Internet]. Singapore: Ministry of Health, Singapore Government; [cited 2020 Oct 9]. Available from: https://www.moh.gov.sg/ifeelyoungsg/how-can-i-age-actively/stay-healthy/project-silver-screen
119. Ministry of Health | Singapore Government. Seniors' Mobility and Enabling Fund [Internet]. [cited 2020 Oct 10]. Available from: https://www.healthhub.sg/a-z/costs-and-financing/26/seniors-mobility-and-enabling-fund-smf
120. Primary Care Pages. Primary Care Network (PCN) [Internet]. Singapore: Agency for Integrated Care; [cited 2020 Oct 9]. Available from: https://www.primarycarepages.sg/practice-management/primary-care-model/primary-care-network-(pcn)
121. The Government of Hong Kong Special Administrative Region. Health Care Voucher [Internet]. Kowloon: The Government of Hong Kong Special Administrative Region; 2022 [cited 2020 Oct 10]. Available from: https://www.hcv.gov.hk/en/index.html
122. Hong Kong Hospital Authority. Chronic Disease Management Project: Risk Assessment and Management [Internet]. Hospital Authority; [cited 2020 Nov 12]. Available from: https://www.ha.org.hk/haho/ho/hacp/RAMP_GOPC_en.pdf
123. Government of Ireland. Treatment Benefit Scheme [Internet]. 2020 [cited 2020 Oct 10]. Available from:

https://www.gov.ie/en/service/1fb655-treatment-benefit-scheme/#
124. National Screening Service Ireland. Diabetic RetinaScreen - The National Diabetic Retinal Screening Programme [Internet]. Health Services Executive; [cited 2020 Oct 10]. Available from: https://www.diabeticretinascreen.ie/
125. Citizens Information Board Ireland. Optometric and ophthalmic services [Internet]. 2019 [cited 2020 Oct 10]. Available from: https://www.citizensinformation.ie/en/health/health_services/dental_aural_and_optical_services/optometric_and_ophthalmic_services.html#
126. HSC Business Serivces Organisation. Free NHS eye tests and optical vouchers [Internet]. National Health Service; 2019 [cited 2020 Oct 11]. Available from: https://www.nhs.uk/using-the-nhs/help-with-health-costs/free-nhs-eye-tests-and-optical-vouchers/
127. HSC Business Services Organisation. Sight Test [Internet]. Business Services Organisation; [cited 2020 Oct 22]. Available from: http://www.hscbusiness.hscni.net/services/1778.htm
128. Welsh Government. Get help with NHS eye care costs [Internet]. Welsh Government; 2020 [cited 2020 Nov 29]. Available from: https://gov.wales/get-help-nhs-eye-care-costs
129. The College of Optometrists. COVID-19 Urgent Eyecare Service (CUES) in England [Internet]. 2020 [cited 2021 Jan 5]. Available from: https://www.college-optometrists.org/the-college/media-hub/news-listing/nhs-england-covid-19-urgent-eyecare-service-cues.html
130. Health and Social Care Board. Northern Ireland Primary Eyecare Assessment and Referral Scheme [Internet]. Belfast: Health and Social Care Board; [cited 2020 Nov 29]. Available from: http://www.hscboard.hscni.net/eyes/
131. Eye Health Examination Wales. Eye Health Examination Wales - including urgent eye care [Internet]. Rhondda Cynon Taff: Wales Eye Care Services; [cited 2020 Oct 22]. Available from: http://www.eyecare.wales.nhs.uk/eye-health-examination-wales
132. Low Vision Service Wales. Low Vision Service Wales [Internet]. Carmarthen: Wales Eye Care Services; [cited 2020 Oct 22]. Available from: http://www.eyecare.wales.nhs.uk/low-vision-service-wales
133. Public Health England & NHS England. Population screening programmes: NHS diabetic eye screening (DES) programme - detailed information [Internet]. gov.uk; [cited 2020 Oct 11]. Available from: https://www.gov.uk/topic/population-screening-programmes/diabetic-eye
134. Public Health Agency. Diabetic Eye Screening Programme (also known as Diabetic Retinopathy Screening) [Internet]. Belfast: Public Health Agency; [cited 2020 Oct 22]. Available from: https://www.publichealth.hscni.net/directorate-public-health/service-development-and-screening/diabetic-eye-screening-programme-also-kn
135. Public Health Scotland. Diabetic retinopathy screening (DRS) in Scotland [Internet]. NHSinform; 2022 [cited 2020 Oct 22]. Available from: https://www.nhsinform.scot/healthy-living/screening/diabetic-retinopathy/diabetic-retinopathy-screening-drs#overview
136. Diabetic Retinopathy Screening Service for Wales. Eye Care Wales - Diabetic Eye Screening Wales [Internet]. Rhondda Cynon Taff: Wales Eye Care Services; [cited 2020 Oct 22]. Available from: http://www.eyecare.wales.nhs.uk/diabetic-retinopathy-screening-service-w
137. Government of the United Kingdom. War Pensioners: Other help you may be entitled to [Internet]. [cited 2020 Oct 22]. Available from: https://www.gov.uk/guidance/war-pensioners-other-help-you-may-be-entitled-to
138. Government of Canada. Guide to vision care benefits [Internet]. 2020 [cited 2020 Oct 15]. Available from: https://www.sac-isc.gc.ca/eng/1579545788749/1579545817396#s1-2
139. Veterans Affairs Canada. Vision Care (POC 14) [Internet]. 2019 [cited 2020 Oct 15]. Available from: https://www.veterans.gc.ca/eng/about-vac/legislation-policies/policies/document/1056
140. Government of Alberta. Health Benefits Card Coverage: 05 Optical Benefits [Internet]. Income and Employment Supports Policy Manual. [cited 2020 Oct 20]. Available from: http://www.humanservices.alberta.ca/AWOnline/HB/4709.html
141. Alberta Government. Dental and Optical Assistance for Seniors [Internet]. [cited 2020 Oct 16]. Available from: https://www.alberta.ca/dental-optical-assistance-seniors.aspx
142. Alberta Government. Alberta Adult Health Benefit [Internet]. [cited 2020 Oct 16]. Available from: https://www.alberta.ca/alberta-adult-health-benefit.aspx
143. Government of British Columbia. Medical Services Plan (MSP) for British Columbia (B.C.) Residents [Internet]. [cited 2020 Oct 15]. Available from: https://www2.gov.bc.ca/gov/content/health/health-drug-coverage/msp/bc-residents
144. Government of Ontario. What OHIP covers [Internet]. [cited 2020 Oct 16]. Available from: https://www.ontario.ca/page/what-ohip-covers#optometry
145. Ministry of Children Community and Social Services | Government of Ontario. Health benefits: Vision [Internet]. [cited 2020 Oct 16]. Available from: https://www.mcss.gov.on.ca/en/mcss/programs/social/odsp/income_support/odsp_vision.aspx
146. Ministry of Health and Long-Term Care | Government of Ontario. Assistive Devices Program [Internet]. [cited 2020 Oct 16]. Available from: http://www.health.gov.on.ca/en/pro/programs/adp/
147. Government of Saskatchewan. Partially Covered Services in Saskatchewan [Internet]. [cited 2020 Oct 16]. Available from: https://www.saskatchewan.ca/residents/health/prescription-drug-plans-and-health-coverage/health-benefits-coverage/partially-covered-services
148. Government of Saskatchewan. Supplementary Health Benefits [Internet]. [cited 2020 Oct 16]. Available from: https://www.saskatchewan.ca/residents/health/prescription-drug-plans-and-health-coverage/extended-benefits-and-drug-plan/supplementary-health-benefits
149. Government of Saskatchewan. Seniors Income Plan (SIP) [Internet]. [cited 2020 Oct 16]. Available from: https://www.saskatchewan.ca/residents/family-and-social-support/seniors-services/financial-help-for-seniors
150. Government of Manitoba. The Manitoba Health Services Insurance Plan [Internet]. [cited 2020 Oct 16]. Available from: https://www.gov.mb.ca/health/mhsip/
151. Government of Manitoba. Health Needs and Other Benefits [Internet]. [cited 2020 Oct 16]. Available from: https://www.gov.mb.ca/fs/eia/eia_benefits.html
152. Government of Manitoba. Seniors Eyeglass Program [Internet]. [cited 2020 Oct 16]. Available from: https://www.gov.mb.ca/health/mhsip/eyeglasses.html
153. Government of Manitoba. Prosthetic Eye Program [Internet]. [cited 2020 Oct 16]. Available from: https://www.gov.mb.ca/health/mhsip/prosthetic_eye.html
154. Government of Nova Scotia. Programs administered through MSI and Nova Scotia Insured Hospital Services [Internet]. 2017 [cited 2020 Oct 16]. Available from: https://novascotia.ca/dhw/msi/docs/MSI_Brochure.pdf

155. Department of Community Services | Government of Nova Scotia. Medical Costs [Internet]. [cited 2020 Oct 16]. Available from: https://novascotia.ca/coms/employment/income_assistance/MedicalCosts.html
156. Department of Community Services | Government of Nova Scotia. Department of Community Services Disability Support Program [Internet]. [cited 2020 Oct 16]. Available from: https://novascotia.ca/coms/disabilities/documents/Disability_Support_Program_Policies.pdf
157. Régie de l'assurance maladie du Québec (RAMQ). Optometric services [Internet]. [cited 2020 Oct 17]. Available from: https://www.ramq.gouv.qc.ca/en/citizens/health-insurance/optometric-services
158. Government of Quebec. Visual aids [Internet]. [cited 2020 Oct 17]. Available from: https://www.ramq.gouv.qc.ca/en/citizens/aid-programs/visual-aids
159. Régie de l'assurance maladie du Québec (RAMQ). Ocular prostheses or artificial eyes [Internet]. [cited 2020 Oct 17]. Available from: https://www.ramq.gouv.qc.ca/en/citizens/aid-programs/ocular-prostheses-artificial-eyes
160. Government of Quebec. Eye examination, glasses or contact lenses [Internet]. [cited 2020 Oct 17]. Available from: https://www.quebec.ca/en/family-and-support-for-individuals/social-assistance-social-solidarity/calculating-basic-benefit/eye-examination
161. Government of New Brunswick. Health Services Vision Program [Internet]. [cited 2020 Oct 17]. Available from: https://www2.gnb.ca/content/gnb/en/services/services_renderer.7975.Health_Services_Vision_Program_.html
162. Government of New Brunswick. Ocular Prosthesis Program [Internet]. [cited 2020 Oct 17]. Available from: https://www2.gnb.ca/content/gnb/en/services/services_renderer.201440.Ocular_Prosthesis_Program.html
163. Government of Prince Edward Island. PEI optometrists now screening for conditions like diabetes [Internet]. [cited 2020 Oct 17]. Available from: https://www.princeedwardisland.ca/en/news/pei-optometrists-now-screening-conditions-diabetes
164. Government of Prince Edward Island. Social Assistance Program [Internet]. [cited 2020 Oct 17]. Available from: https://www.princeedwardisland.ca/en/information/family-and-human-services/social-assistance-program
165. Immigration Skills and Labour | Government of Newfoundland Labrador. Health Related Services [Internet]. [cited 2020 Oct 17]. Available from: https://www.gov.nl.ca/isl/income-support/healthservices/
166. Department of Health and Social Services | Government of Northwest Territories. NWT Métis Health Benefits Program [Internet]. [cited 2020 Oct 17]. Available from: https://www.hss.gov.nt.ca/en/services/supplementary-health-benefits/metis-health-benefits
167. Health and Social Services Authority | Government of Northwest Territories. Eye Clinic – Ophthalmology Services [Internet]. [cited 2020 Oct 17]. Available from: https://www.nthssa.ca/en/services/eye-clinic-—-ophthalmology-services#
168. Government of Yukon. Extended health care benefits to seniors [Internet]. [cited 2020 Oct 17]. Available from: https://hss.yukon.ca/extendedcare.php
169. Medicare. Medicare.gov: the official U.S. government site for Medicare | Medicare [Internet]. [cited 2021 Jan 6]. Available from: https://www.medicare.gov/
170. Centres for Medicare & Medicaid services. Medicaid.gov: the official U.S. government site for Medicare | Medicaid [Internet]. [cited 2021 Jan 7]. Available from: https://www.medicaid.gov/
171. U.S. Department of Veterans Affairs. VA Vision Care | Veterans Affairs [Internet]. [cited 2021 Jan 7]. Available from: https://www.va.gov/health-care/about-va-health-benefits/vision-care/
172. Tricare. Home | TRICARE [Internet]. [cited 2021 Jan 7]. Available from: https://tricare.mil/
173. U.S. Department of Veterans Affairs. CHAMPVA Benefits | Veterans Affairs [Internet]. [cited 2021 Jan 7]. Available from: https://www.va.gov/health-care/family-caregiver-benefits/champva/
